# Machine Learning Reveals Missing Edges and Putative Interaction Mechanisms in Microbial Ecosystem Networks

**DOI:** 10.1128/mSystems.00181-18

**Published:** 2018-10-30

**Authors:** Demetrius DiMucci, Mark Kon, Daniel Segrè

**Affiliations:** a Bioinformatics Graduate Program, Boston University, Boston, Massachusetts, USA; b Biological Design Center, Boston University, Boston, Massachusetts, USA; c Department of Mathematics and Statistics, Boston University, Boston, Massachusetts, USA; d Department of Biology, Boston University, Boston, Massachusetts, USA; e Department of Biomedical Engineering, Boston University, Boston, Massachusetts, USA; f Department of Physics, Boston University, Boston, Massachusetts, USA; Heidelberg University

**Keywords:** coculture experiments, ecological networks, flux balance analysis, machine learning, metabolic modeling, microbial interactions, microbiome, random forests, synthetic ecology, systems biology

## Abstract

Microbes affect each other’s growth in multiple, often elusive, ways. The ensuing interdependencies form complex networks, believed to reflect taxonomic composition as well as community-level functional properties and dynamics. The elucidation of these networks is often pursued by measuring pairwise interactions in coculture experiments. However, the combinatorial complexity precludes an exhaustive experimental analysis of pairwise interactions, even for moderately sized microbial communities. Here, we used a machine learning random forest approach to address this challenge. In particular, we show how partial knowledge of a microbial interaction network, combined with trait-level representations of individual microbial species, can provide accurate inference of missing edges in the network and putative mechanisms underlying the interactions. We applied our algorithm to three case studies: an experimentally mapped network of interactions between auxotrophic Escherichia coli strains, a community of soil microbes, and a large *in silico* network of metabolic interdependencies between 100 human gut-associated bacteria. For this last case, 5% of the network was sufficient to predict the remaining 95% with 80% accuracy, and the mechanistic hypotheses produced by the algorithm accurately reflected known metabolic exchanges. Our approach, broadly applicable to any microbial or other ecological network, may drive the discovery of new interactions and new molecular mechanisms, both for therapeutic interventions involving natural communities and for the rational design of synthetic consortia.

**IMPORTANCE** Different organisms in a microbial community may drastically affect each other’s growth phenotypes, significantly affecting the community dynamics, with important implications for human and environmental health. Novel culturing methods and the decreasing costs of sequencing will gradually enable high-throughput measurements of pairwise interactions in systematic coculturing studies. However, a thorough characterization of all interactions that occur within a microbial community is greatly limited both by the combinatorial complexity of possible assortments and by the limited biological insight that interaction measurements typically provide without laborious specific follow-ups. Here, we show how a simple and flexible formal representation of microbial pairs can be used for the classification of interactions via machine learning. The approach we propose predicts with high accuracy the outcome of yet-to-be performed experiments and generates testable hypotheses about the mechanisms of specific interactions.

## Abstract (precis)

Different organisms in a microbial community may drastically affect each other’s growth phenotypes, significantly affecting the community dynamics, with important implications for human and environmental health. Novel culturing methods and the decreasing costs of sequencing will gradually enable high-throughput measurements of pairwise interactions in systematic coculturing studies. However, a thorough characterization of all interactions that occur within a microbial community is greatly limited both by the combinatorial complexity of possible assortments and by the limited biological insight that interaction measurements typically provide without laborious specific follow-ups. Here, we show how a simple and flexible formal representation of microbial pairs can be used for the classification of interactions via machine learning. The approach we propose predicts with high accuracy the outcome of yet-to-be performed experiments and generates testable hypotheses about the mechanisms of specific interactions.

## INTRODUCTION

The collective behavior of microbial ecosystems across biomes is an outcome of the many interactions between members of the community (1–7). These interactions include the exchange of metabolites, signaling and quorum sensing processes, as well as growth inhibition and killing. An understanding of the interspecific interactions within microbial communities is essential for understanding the function of natural ecosystems (1–3, 6, 8) and for the design of synthetic consortia (5, 9–12).

A powerful and increasingly employed method for assessing microbial interactions is the direct measurement of phenotypes of microbial species grown in coculture (12, 13). A fundamental challenge in this endeavor is the huge diversity of many natural communities, which may include up to several hundred strains or species of microbes. Performing the experiments for all possible pairwise interactions constitutes a herculean and likely insurmountable task for even a moderately sized community. However, it is conceivable that new computational approaches could systematically complement existing tools such as high-throughput sequencing and genome annotation (14–18) to help extract as much information as possible from interaction data sets, providing insight both on yet-to-be-measured interactions and on the possible biological mechanisms mediating the specific partnerships.

Here, we present a conceptual framework for the mathematical representation of microbial interactions and the subsequent use of supervised learning to build a classifier with high predictive accuracy. While any algorithm may be used, we obtained our best results with a random forest algorithm (19–21). Random forests are ensembles of many decision trees that individually are poor classifiers but can be pooled to create a very good classifier. Random forests have two attributes that we found particularly attractive for our purposes. First, they are nonparametric and thus require no *a priori* definitions or assumptions about the underlying relationships between predictive variables. Second, recent methodological developments in the interpretation of random forests were made that enable users to query why specific examples are classified as they are, through the calculation of feature contributions (22). The feature contributions can be exploited to develop new hypotheses about the mechanisms that mediate specific interactions. To demonstrate a proof of principle for the classification of microbial interactions using organism traits and the utility of feature contributions for developing insight into the underlying mechanisms, we applied this approach to three communities where all pairwise experiments had been performed. The first was an *in silico* community of 100 metabolic models of human gut-associated bacteria. The second community involved 14 strains of amino acid auxotrophic Escherichia coli. The third community was a collection of 20 microbial strains that were isolated from the same soil sample. Our results show that the combination of random forests with trait-level representations resulted in high-performance classifiers. Furthermore, feature contributions have the potential to facilitate the discovery of new interaction mechanisms.

## RESULTS

### Representing pairwise interactions.

**FIG 1:**
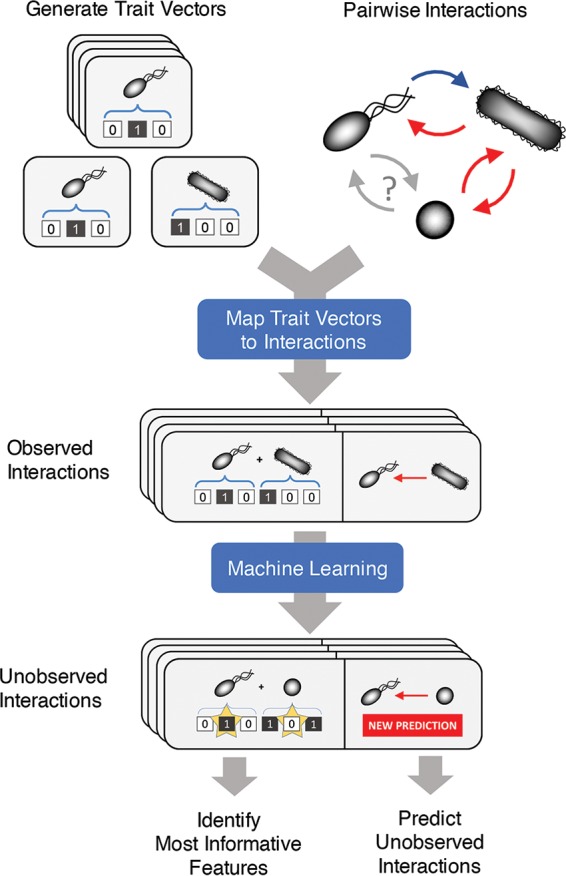
Schematic representation of our machine learning approach for inferring interactions among microbes. A trait vector captures the characteristics of each organism in the community of interest. The presence or absence of a trait in a given organism is encoded (as a binary number) in the corresponding element of the trait vector. For every possible pairwise interaction among community members, we construct a composite vector that is the concatenation of the corresponding trait vectors. The vector of the organism whose response is being predicted is concatenated to the front of the trait vector of its interaction partner. For the set of observed interactions, each composite vector is then mapped to the measured response of the interacting species. All observed interactions are then used to train a model that predicts the outcome of unobserved interactions. If random forest is used, then feature contributions can be calculated on a case-by-case basis to identify which elements of the composite genome contribute most strongly to the prediction.

Our objective in this study was twofold. First, we sought to predict the qualitative outcomes of unobserved pairwise interactions in microbial communities. Second, we wanted to identify predictive variables that suggest potential mechanisms of interaction. To achieve both of these goals, it was important to establish a representation that can be used by an algorithm to make good predictions and can also be easily parsed for interpretation. Our approach relies on the availability of trait-level descriptions for each organism in the community under consideration. These trait descriptions were used to construct feature vectors for each organism (see Materials and Methods). Specific interactions are represented as the concatenation of the relevant trait vectors (Fig. 1). Trait vectors may be constructed from any set of biologically relevant features, such as the presence/absence of a certain gene or metabolic function, phylogenetic classifications, or even characteristics of the environment where the organism was found. In our analyses, different case studies were based on different trait vector representations: in particular, we used (i) the presence/absence of metabolic reactions for the *in silico* community case study, (ii) binary vectors of biosynthetic capabilities for each E. coli strain in the auxotroph community case study, and (iii) metabolic functions predicted from 16s sequences for the soil community case study.

These trait vectors, together with the known outcome of a subset of interactions, can be fed into machine learning algorithms that separate outcome classes and subsequently predict the outcome of unobserved interactions. Here, we used the random forest algorithm, based on an ensemble of many decision trees that individually ask a series of yes or no questions about randomly selected subsets of predictive features to classify samples. To find potential mechanisms of interaction, we took advantage of the structure of individual trees to identify which variables are the most influential for the classification of specific samples.

### Application to computationally predicted interactions between human gut microbes.

We first applied our approach to a large *in silico* data set generated by simulating time course microbial coculture experiments with dynamic flux balance analysis (23, 24) using Computation of Microbial Ecosystems in Time and Space (COMETS) (5) (see Materials and Methods). The dynamic flux balance analysis enables the computation of approximate growth curves on the basis of the complete metabolic networks of the microbes (derived from their sequenced genomes) and the abundance of each nutrient present in the medium at the beginning of the experiment. The simulated experiment provides an estimate of the final biomass for each organism and the exchange fluxes during exponential growth. The possible interactions between different species in the coculture may result from the exchange of secreted by-products or the competition for common nutrients. To generate a large set of observations for machine learning, we selected metabolic models of 100 human gut-associated bacteria (25) and used COMETS to simulate all pairwise coculture interactions within the same rich medium in a well-mixed batch culture scenario.

**FIG 2:**
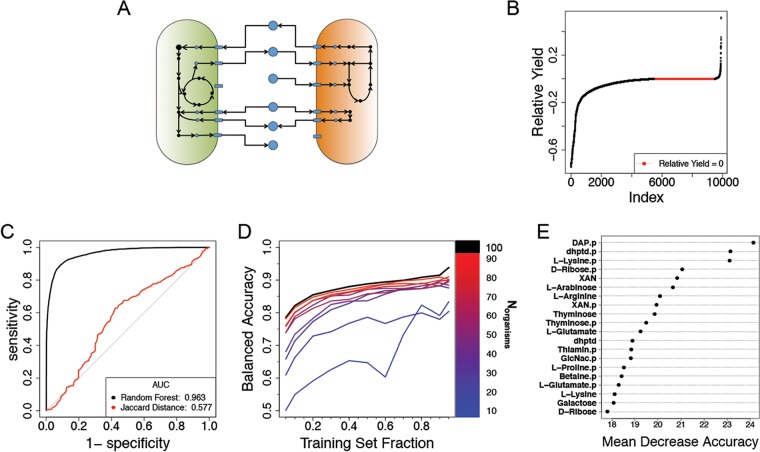
Classification of pairwise interactions for an *in silico* model of a community of human gut microbes. (A) Organisms are represented *in silico* as large networks of metabolic reactions that take up metabolites (blue circles) from the environment (arrows leading to model) and release by-products (arrows leading to metabolite). Organisms may interact with one another during the simulation when both organisms compete for the uptake of a metabolite or through cross feeding, where one model consumes a by-product of the other. (B) Relative yields from all experiments are plotted in ascending order. There were 5,563 samples with a negative relative yield. Neutral interactions, a relative yield of zero, occurred 3,917 times, and positive relative yield occurred 420 times. Samples were classified as negative or nonnegative. (C) For all 9,900 *in silico* observations, the ROC curve of a random forest classifier was determined by using 388 exchange reactions as predictors and compared to the ROC curve obtained from using the Jaccard distance as a simple threshold to predict negative versus nonnegative relative yields. Values for the ROC curve were obtained by evaluating the class voting ratios on out-of-bag samples (see Materials and Methods). ROC curves for classifiers trained on subsets of the data can be seen in Fig. S3 in the supplemental material. (D) Learning curves for subcommunities of the full *in silico* community. These learning curves are the median learning curves evaluated with 10-fold cross-validation on test sets at each point (see Materials and Methods) for 5 subcommunities selected at random for each value of *N*_organisms_. (E) A representation of the 20 most influential predictors as determined by mean decrease in accuracy. Labels on the *y* axis indicate the feature names. A “p” suffix in the label indicates that the predictor is a feature of the interaction partner; DAP, meso-2,6-diaminopimelate; dhptd, 4,5-dihydroxy-2,3-pentanedione; XAN, xanthine; GlcNac, *N*-acetylglucosamine. The results of an alternative representation scheme using phylogenies are presented in Fig. S5.

The trait vectors used to represent each organism were simply binary vectors indicating the presence or absence of various nutrient exchange reactions in the metabolic network models (see Materials and Methods and Fig. 2A). The interactions in the network were computed by determining the influence of every organism on every other organism in COMETS coculture simulations. In particular, the simulations provided the final biomass of each organism in coculture and in monoculture. A normalized difference between these two yields (i.e., the relative yield; see Materials and Methods) was used as the phenotypic metric for classifying the interaction (negative or nonnegative) (Fig. 2B).

The random forest algorithm was first applied to the full data set, revealing that its out-of-bag (OOB) accuracy (roughly equivalent to a 5-fold cross-validation; see reference 26 and Materials and Methods) was approximately 90.5%. The receiver operator characteristic (ROC) curve for the random forest algorithm (Fig. 2C and Materials and Methods) compares favorably to a naive prediction based on the Jaccard distance (27) between the different trait vectors (see Materials and Methods and see references 28 and 29 for similar use of Jaccard distance in microbial community studies).

The high predictive accuracies are encouraging but are of little use if they can only be achieved when the vast majority of the experiment outcomes are already known. Thus, we constructed a series of learning curves to visualize how the balanced accuracy of the random forest classifier is affected by the size of the community and by the amount of training data available (Fig. 2D). For small communities (for example, *N*_organisms_ = 10), there is little gain in predictive performance until the experimental space is nearly totally known. However, when *N*_organisms_ is increased to 20 (which amounts to 190 pairwise experiments, corresponding to 380 individual responses to coculture), as little as 5% of the total data (∼9 to 10 experiments, i.e., 18 to 20 responses) is sufficient to obtain useful predictions. The ROC curves and comparison with a Jaccard distance classifier for selected points along the learning curve showed a similar trend to what seen for the full data set (see Fig. S3 in the supplemental material). The general trend indicates that the larger a community is, the smaller the relative fraction of experiments needed to obtain a high accuracy. In general, learning curves can be used as guidelines to determine how many experiments should be implemented to reach a target performance.

**FIG 3:**
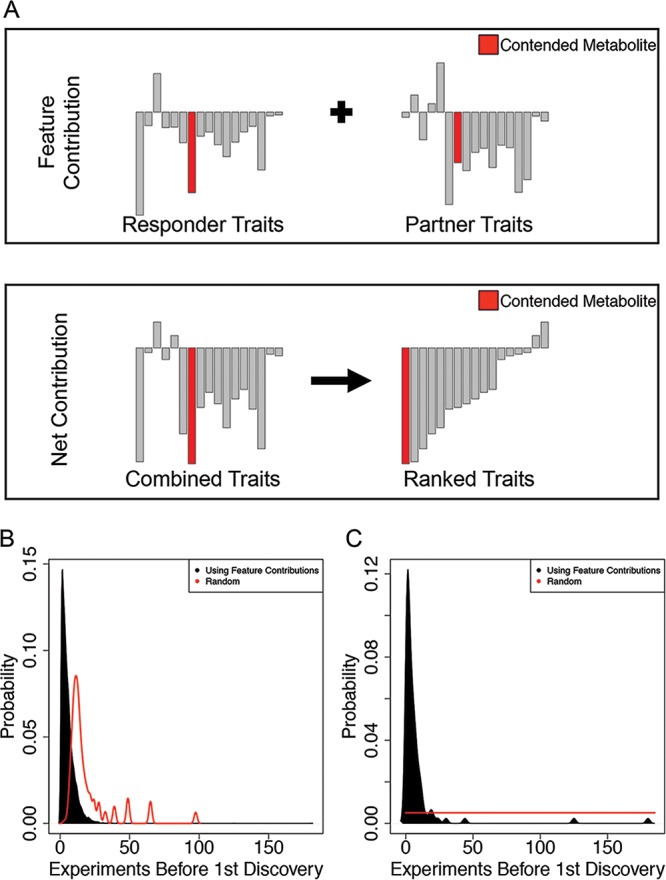
Using feature contributions to find a metabolite for which two organisms compete. (A) Metabolite transporters belonging to the organism of interest (top left) or the interaction partner (top right). We were interested in identifying a metabolite that is associated with the negative relative yield for the organism of interest. To establish a ranking of metabolites, the feature contributions from both of the composite trait vectors (top) were summed and sorted according to the net contribution (bottom). Proceeding from the negative end, the rank and identity of the first contended metabolite encountered relative to the negative end of the new vector was recorded. (B) The probability distributions of the average rank at which the first mechanistic metabolite is encountered by sampling metabolites randomly one at a time calculated for each sample and for feature contributions. By chance, the first metabolite is encountered after 13 random queries. Feature contributions reduce the median number of queries to 4. (C) Ninety-nine samples produced a negative relative yield through the competition for one metabolite. Randomly investigating each of the 194 candidate metabolites results in an average of 97.5 experiments before discovering the metabolite. By using feature contributions to prioritize the order in which to investigate metabolites, the contended metabolite is revealed on or before the fourth experiment (median = 4).

In addition to confirming that the algorithm accurately classifies unobserved interactions, we investigated whether the top feature vector components used as the predictors are biologically interpretable. The variable importance plot (see Materials and Methods and Fig. 2E) shows the globally most informative trait vector components. In this case, the most important predictor for the classification of a given organism was a feature of the interaction partner (Fig. 2E). In other words, the predicted growth phenotype of organism *i* in the presence of organism *j* is best described by features that are in the vector for organism *j*. In addition to analyzing the global contributions of variables to the classifications across all data, the tree-based approach of random forests can be used to determine why specific samples were classified as they were by examining the feature contributions for specific interactions. A feature contribution (see details in Materials and Methods) quantifies how much a given variable typically influences the classification probability of a single sample. Feature contributions were originally developed for the analysis of regression models (30) but have since been adapted for binary classification models (22). We wondered whether the simulated data could be used to illustrate the possible value of feature contributions for identifying putative biological mechanisms underlying a given interaction. In particular, we envisaged that the random forest algorithm, trained only on the basis of the trait profiles and the relative yields in cocultures, could be used to suggest which metabolites may be more likely to mediate a given competitive (Fig. 3) or facilitative (see Fig. S1) interaction. As opposed to an *in vitro* system, where such a prediction would need to be validated with new experiments, our *in silico* system enables the value of the random forest prediction to be checked by comparing it with simulated exchange fluxes across the two species (which, importantly, were not used in training the random forest).

10.1128/mSystems.00181-18.1 FIG S1 Feature contributions were used to find a facilitative metabolite in samples that had a positive relative yield (RY > 0). The empirical probability distribution of the average rank at which the first facilitative metabolite would be encountered by sampling metabolites randomly one at a time was calculated for each sample and compared to the observed probability distribution obtained from using ranked feature contributions. By chance, the median first metabolite is encountered after 65 queries (mean ≈ 58.7). With feature contributions, the median number of queries was 27 (mean ≈ 50). The number of experiments required by chance to find the first metabolite in a sample is a function of the number of real mechanisms in that sample and is the cause of the observed multimodality. Positive samples were scarce in the *in silico* data set (420/9,900). A reliable classifier was developed via a balanced training set created by randomly sampling 420 nonpositive samples. This process was repeated 100 times, with an observed median balanced accuracy of ∼85%. A single random forest model was then used to calculate the feature contributions for the identification of putative facilitative metabolites. Download FIG S1, PDF file, 0.02 MB. Copyright © 2018 DiMucci et al. 2018 DiMucci et al. This content is distributed under the terms of the Creative Commons Attribution 4.0 International license.

Towards this goal, for each pair of organisms, we ranked—by their net feature contributions (see Materials and Methods, Fig. 3A, and Table S1)—the 194 metabolites involved in exchange reactions. We found that the metabolites ranking highly on the basis of this criterion were much more likely than random to be among the metabolites truly exchanged in the COMETS simulations (Fig. 3B). This is particularly valuable if the interaction is due to a single exchanged metabolite (Fig. 3C). In practice, if this criterion was used on *in vitro* data, it would imply a significant reduction in the number of tests needed to identify at least one mechanism of interaction.

10.1128/mSystems.00181-18.6 TABLE S1 Top metabolites for which pairs of organisms are predicted to compete based on ranked feature contributions. Download Table S1, PDF file, 0.04 MB. Copyright © 2018 DiMucci et al. 2018 DiMucci et al. This content is distributed under the terms of the Creative Commons Attribution 4.0 International license.

It is also instructive to look in more detail at a specific case of feature contribution analysis. In particular, we observed that fructose exchange was most frequently the strongest predictor of competitive interactions (it was the top ranking true feature in ∼18.7% of all competitive interactions) (Table S1), and it corresponded to the 15th most common true mechanism based on the COMETS-simulated fluxes (see Table S2). Interestingly, fructose has been implicated in altering the gut microbiome in a number of diseases, including antibiotic-treatable metabolic syndrome (31–33), liver disease (34), and obesity (35). Our approach is also readily applicable for the discovery of metabolites that mediate positive interactions, which comprise a small minority of all interactions (420/9,900). Due to the scarcity of their occurrence and the dearth of metabolites that mediate positive interactions, the discovery of these mechanisms is more challenging. Nevertheless, the use of ranked feature contributions to find the facilitative metabolites was a powerful improvement over a naive approach (Fig. S1).

10.1128/mSystems.00181-18.7 TABLE S2 Numbers of times each of the 194 metabolites were consumed by both organisms in negative interactions. Metabolite cpd00082 corresponds to fructose. Legends for all metabolites can be found at http://modelseed.org/biochem/compounds. Download Table S2, PDF file, 0.02 MB. Copyright © 2018 DiMucci et al. 2018 DiMucci et al. This content is distributed under the terms of the Creative Commons Attribution 4.0 International license.

### Application to a community of auxotrophic Escherichia coli strains.

**FIG 4:**
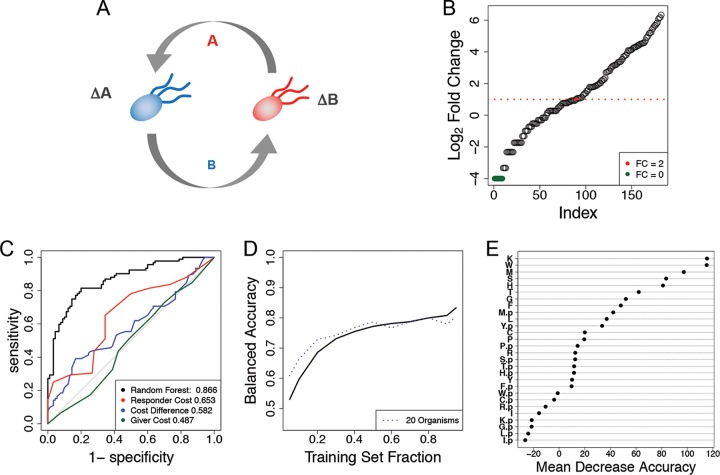
Data representation and results for the case study of a network of auxotrophic E. coli strains. (A) In the original experiment, single-gene knockout E. coli auxotrophs were cocultured in minimal medium. For the ΔA mutant to grow, it must receive amino acid A from the ΔB mutant, which in turn must receive another amino acid, B, for growth. Auxotroph strains were constructed for the following amino acids: cysteine, phenylalanine, glycine, histidine, isoleucine, leucine, methionine, proline, arginine, serine, threonine, tryptophan, and tyrosine. (B) Auxotroph strain fold changes in ascending order. E. coli strains had a weak response (fold change ≤ 2) 90 times and failed to grow 9 times (green circles). In 92 instances, the E. coli auxotroph population more than doubled over the course of 84 h. (C) ROC curves for all 182 observations were determined for a random forest classifier using 28 amino acids as predictors. Single-value thresholds based on the biosynthetic costs of knocked-out amino acids resulted in poorer performance than the random forest algorithm. (D) Trajectory of a learning curve built for the E. coli interactions (solid line) closely resembles that of the learning curve for *in silico* communities with 20 organisms (dashed line). (E) Ranking of 28 amino acids according to their effects on prediction accuracy when randomly permuted. Amino acids corresponding to the receiver strain are enriched near the top of the list. A suffix “p” indicates that the predictive feature belongs to the giver strain. A single case of a Δmethionine mutant cocultured with a Δcysteine mutant is shown in Fig. S2.

We next applied the random forest algorithm to experimental data on auxotrophic E. coli cocultures. In particular, we used previously published data from all possible cocultures of 14 E. coli strains, each auxotrophic for a given amino acid (36). The interactions between any given pair of E. coli strains are presumably dependent on the direct exchange of the missing amino acids or related precursors (Fig. 4A). The total growth of each strain in the 91 experiments was measured after 84 h and reported as the net fold change relative to the initial inoculum, resulting in 182 total observations (see Materials and Methods for additional comments on the experimental setup). We built trait vectors according to the 14 amino acids and labeled growth phenotypes according to the fold change response of a given E. coli auxotroph strain in coculture with another auxotrophic strain (using 2 as the fold change cutoff for distinguishing between “strong” and “weak” interaction phenotypes) (Fig. 4B).

10.1128/mSystems.00181-18.2 FIG S2 (A) Bar plot of the calculated feature contributions for the growth response of a ΔMet mutant cocultured with a ΔCys mutant in the E. coli auxotrophs case study. The feature contribution from the receiver methionine is ≈0.41. The feature contribution from the giver cysteine is ≈−0.03. The net contribution from the remaining predictors is ≈0.02. (B) The ΔMet mutant typically had a strong response in coculture no matter the identity of its interaction partner; 12 interactions resulted in strong response type for the ΔMet mutant. When it was grown with a ΔCys mutant, however, it had a weak growth response. The use of feature contributions correctly identified the receiver’s methionine and the giver’s cysteine as the first and second most important predictors, respectively, in this interaction. The contribution from the receiver methionine is overwhelmingly positive, reflecting the fact that the ΔMet mutant typically benefits strongly from coculture and results in the strong response prediction in panel A. To develop a hypothesis for why this interaction defied the expectations of the random forest, we consulted the literature regarding the biosynthetic pathways for methionine and cysteine and learned that under the specified growth conditions, cysteine is necessary for the biosynthesis of cystathionine. Cystathionine is subsequently required for the biosynthesis of homocysteine, which is in turn required for the production of methionine. Given this knowledge, we suspect the ΔCys mutant is unable produce methionine until enough cysteine accumulates in the environment, and the ΔMet mutant must wait for the ΔCys mutant to use its excess cysteine for the production of excess methionine (B). The ΔMet mutant is likely not able to provide an abundance of extracellular cysteine for the ΔCys mutant early in the interaction, because cysteine biosynthesis is tightly regulated due to its toxicity, but (i) efflux of cysteine has been proposed as a potential regulatory mechanism and (ii) likely enables the ΔMet mutant to produce low levels of extracellular cysteine. The waiting time associated with ΔMet mutant-derived cysteine to accumulate in the environment would result in delayed growth for both strains relative to other interactions involving the ΔMet mutant. The reported fold changes in this experiment were 0.8 for the ΔMet mutant and 1.2 for the ΔCys mutant. The weak growth response of the ΔMet mutant is consistent with delayed growth. Download FIG S2, PDF file, 0.03 MB. Copyright © 2018 DiMucci et al. 2018 DiMucci et al. This content is distributed under the terms of the Creative Commons Attribution 4.0 International license.

The random forest algorithm yielded a balanced accuracy of ∼79.2% in predicting this interaction phenotype. An examination of the corresponding ROC curves showed that the random forest is a much better predictor than simpler metrics based on biosynthetic costs (36) of the different amino acids (Fig. 4C). The learning curve for this test case (Fig. 4D) resembles the trajectory of the learning curve for *in silico* communities of 20 members (Fig. 2D). The variable importance rankings show that, in general, the amino acid needed by the receiver has a greater impact on the classification accuracy than the amino acid its partner needs, suggesting that the specificity of the interaction is dominated by auxotrophies, whereas most mutants can, in principle, provide the missing amino acid (Fig. 4E).

As done for the *in silico* simulations, we next analyzed the feature contributions and asked whether they reflect the underlying mechanisms. In particular, we asked how often the absence of one of the two amino acids for a pair of organisms has the strongest contribution in the random forest algorithm. As expected, the random forest algorithm is more strongly influenced by the absence of an amino acid feature than by its presence. Of all 182 observations, the absence of the amino acid from the receiver had the largest feature contribution 140 times, and the absence of the amino acid from the giver had the largest contribution 40 times (see Table S3). Thus, the pair of most influential predictors tended to correspond to the underlying mechanism of the interaction, even in instances where the predicted class was incorrect. Scenarios where the presumed mechanisms are the strongest contributors sometimes resulted in misclassification, presenting opportunities for direct research of interesting outliers. The response of the methionine auxotroph (ΔMet mutant) in coculture with the cysteine auxotroph (ΔCys mutant) was one such case, which we describe in detail in Fig. S2.

10.1128/mSystems.00181-18.8 TABLE S3 Counts of how often one or both auxotrophic amino acids were the strongest predictors for *E. coli* interactions. Download Table S3, PDF file, 0.04 MB. Copyright © 2018 DiMucci et al. 2018 DiMucci et al. This content is distributed under the terms of the Creative Commons Attribution 4.0 International license.

### Application to a community of soil bacteria.

**FIG 5:**
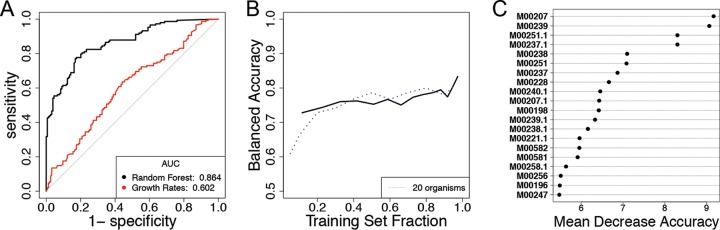
(A) ROC curves from the random forest trained on all 302 observations using 79 predicted KEGG modules as features. The difference in the initial growth rates of both strains was used as a baseline simple predictor. (B) The learning curve built on this data set starts at ∼72% balanced accuracy and tops out at ∼78% balanced accuracy (solid line). The learning curve for the *in silico* communities with 20 organisms is displayed for comparison (dashed line). (C) The identifiers (IDs) of the most important modules for predictive accuracy of the forest. See Table S4 for the full module names.

For the final test case, we analyzed the results of a study featuring all pairwise coculture experiments of 20 bacterial strains isolated from the same soil sample (3) (see Materials and Methods). For each experiment, the authors reported whether each species was present at a detectable level at the final time point. We built trait vectors according to the presence or absence of KEGG modules (37) as predicted by PICRUSt (38). The random forest algorithm trained on the full data set provided an out-of-bag balanced accuracy of 79.4%. The ROC curve shows that the random forest algorithm performed much better than a simple decision rule based on the differences in the reported initial growth rates of each species (Fig. 5A). The learning curve for this community closely resembles that of the 20-member communities from our *in silico* case study (Fig. 5B). The variable importance plot shows that the predictions were most strongly influenced by the transport of teichoic acids (which are found in the walls of several Gram-positive bacteria [39]), both in the strain being predicted and in its interaction partner (Fig. 5C, see also Table S4 for KEGG module names). Further insight into relevant pair-specific KEGG modules can be obtained from feature contributions (see Table S5).

10.1128/mSystems.00181-18.9 TABLE S4 KEGG module names and IDs identified with PICRUSt for the soil bacteria case study dataset. Download Table S4, PDF file, 0.03 MB. Copyright © 2018 DiMucci et al. 2018 DiMucci et al. This content is distributed under the terms of the Creative Commons Attribution 4.0 International license.

10.1128/mSystems.00181-18.10 TABLE S5 Functional modules (and the types of predictions they contribute) associated with the most informative features that describe different pairwise interactions in the soil bacteria case study. Download Table S5, PDF file, 0.1 MB. Copyright © 2018 DiMucci et al. 2018 DiMucci et al. This content is distributed under the terms of the Creative Commons Attribution 4.0 International license.

## DISCUSSION

Exhaustive pairwise coculture studies of microbial strains are an increasingly common avenue for estimating an ecosystem interaction network. While such pairwise interactions do not necessarily capture all possible interdependencies in a community (4, 40), they have been shown to be a dominant factor (12), making the reliable prediction and interpretation of predictive models matters of great importance. In this study, we described a conceptual framework for the representation of microbes and their pairwise interactions to address both of these challenges.

The ideal data sets for testing our approach would include a large number of pairs of microbes and genotypes or multidimensional phenotypes for each species. While we envisage that a multitude of such data sets will be available in the future, the existing data sets are either limited in size or in trait vector accessibility. Thus, we tested our approach on three data sets, each with a different set of advantages and limitations. The first and largest data set was obtained by simulating 4,950 microbial cocultures with dynamic flux balance metabolic modeling (COMETS). An important caveat about this specific test case is that metabolic models may not capture the full biochemical details of the real system they approximate, and they do not incorporate any of the nonmetabolic processes that may be observed in real communities (41). However, these models have been used to successfully help understand the physiology of specific organisms (42) and communities (5, 43, 44). The other two experimental studies we used were not affected by these issues, but they were limited in the numbers of organisms and pairs analyzed. The first experimental data set was the outcome of a study involving 14 strains of E. coli amino acid auxotrophs. In this case, the trait vectors were a straightforward representation of the auxotrophies, but the random forest algorithm highlighted the complexity of the underlying interdependencies. The second experimental data set was from a community of soil microbes, whose trait vectors were derived from the available 16s rRNA sequences, suggesting a broad applicability of our approach to future similar studies.

The qualitative prediction of the outcome of unobserved interactions is most valuable if that prediction leads to a reduction in the usage of precious resources and time. To this end, the construction of learning curves is an important step in identifying how much data are required to achieve the desired prediction accuracy from machine learning. This may be particularly useful for planning large-scale studies of naturally cooccurring species or synthetic consortia, e.g., for searching communities with specific properties relevant for biomedical or engineering applications (45).

Despite the common perception that random forest algorithms are merely uninterpretable “black boxes,” we showed here that feature contributions provide a clear window into the decision-making process of a random forest. If the features are defined on the basis of clearly identifiable biological entities (e.g., genes, reactions, or phenotypic traits), then the feature contributions can be effectively used to guide experiments that help reveal the underlying mechanisms.

In the current implementation of our algorithm, we concatenated the binary trait vectors of two organisms to form a new composite trait representation. However, alternative representations of microbes and their interactions are possible and should be explored. These may also include more quantitative information, such as the gene copy number or the mean transcriptional levels. While in our current work the environment for each case study was fixed, it is also possible to apply our method to data from heterogeneous environments, provided that the environmental parameters are encoded in the trait vector.

The present study focused entirely on demonstrating the possible benefits of applying machine learning to the study of interspecies interactions in microbial communities. In this context, our use of mechanistic models (based on dynamic flux balance analysis) was limited to the generation of *in silico* data sets meant to enable the testing of our approach. However, we envisage that in the future it will be possible to integrate machine learning and mechanistic approaches toward a better characterization and design of microbial consortia. More broadly, we foresee that the interplay of quantitative approaches with high-throughput genotypic and phenotypic measurements will constitute a very valuable instrument for future microbiome research and synthetic ecology.

## MATERIALS AND METHODS

### Representation of interactions with trait-derived features.

For a given community *C*, the observed coculture response of each species *i* in the presence of species *j* is encoded in the element *X_ij_* of a community matrix *X*. *X_ij_* represents the appropriately normalized abundance of species *i* at the end of a coculture experiment with species *j* or a binary variable describing whether or not species *i* will survive after inoculation with species *j*. To define a set of trait vectors for each organism in *C*, a list of *n* features was obtained that can be assigned systematically across all organisms. These features include the presence/absence of specific genes, metabolic functions, or any other relevant trait, provided these features are not dependent on or derived from the quantities being measured. Thus, each organism *i* is assigned an *n*-long vector, *F*^*(i)*^, such that *F*_*k*_^*(i)*^ is 0 or 1 depending on whether the corresponding trait is absent or present, respectively, in the organism. Each pair of organisms (*i*,*j*) is then associated with a coculture feature vector, defined as the concatenation of vectors *F*^*(i)*^ and *F*^*(j)*^ indicated as *F*^*(i,j)*^ = [*F*^*(i)*^, *F*^*(j)*^] (see Fig. 1). The behavior of a specific organism from a pair in coculture is thus formally described by the concatenated feature vector *F*^*(i,j)*^ and the corresponding phenotype *X*_*ij*_. Note that in general, *F*^*(i,j)*^ ≠ *F*^*(j,i)*^ and *X*_*ij*_ ≠ *X*_*ji*_.

### Data generation for case study of *in silico* gut microbe interactions.

Metabolic reconstructions of human gut-associated microbes were obtained from Bauer et al. (25). At the time of this writing, these models were available for download at https://wwwen.uni.lu/content/download/86230/1056013/file/Bauer_et_al_301_microbe_models.rar.

Each metabolic reconstruction encompassed the stoichiometry of virtually all metabolic reactions present in an organism, including uptake/secretion. Flux balance analysis (FBA) is a constraint-based steady-state approach that uses this stoichiometry to predict fluxes and growth capacity under a given boundary condition of nutrient availability and has been described in detail (24, 41, 43, 46). Briefly, the set of reactions contained in a model is derived from the organism’s genome annotation. The reactions are then used to construct the stoichiometric matrix *S* for the metabolic model, whose element *S_ij_* indicates the number of molecules of type *i* used or produced by reaction *j*. The identification of feasible metabolic fluxes (v) for the system is achieved by imposing a steady state (*S*v = 0), as well as upper/lower bound constraints that define the environmental nutrient availability. Standard flux balance analysis calculations then use linear optimization to identify feasible flux states that maximize a given objective function, usually the growth flux of the cell, i.e., the production of a balanced biomass composition of the organism.

Dynamic flux balance analysis (dFBA) (23) extends the classical FBA to perform dynamic simulations in which intracellular metabolites are still assumed to be at steady states but total biomass and environmental metabolites are treated as time-dependent variables in a discretized approximation. Crucially, in a dFBA simulation of multiple species, competition or facilitation (e.g., cross feeding) are emergent properties of the flux dynamics of individual organisms. Thus, no *a priori* assumptions need to be made about the existence or nature of the ecological interactions. dFBA simulations were performed using our platform for Computation of Microbial Ecosystems in Time and Space (COMETS), which was previously used to model microbial communities (5). One hundred metabolic models (25) were selected, and a common medium that enables the growth of nearly all models in a monoculture scenario was identified. Pairwise coculture simulations of the 100 models were performed by using the common medium in a well-mixed batch culture scenario (approximated by using COMETS without spatial structure). For each scenario, the biomass accumulation and fluxes were used to calculate the relative yield and identify the mechanisms of interaction, respectively. In this case, *X_ij_* corresponds to the relative yield of strain *i* in coculture with strain *j* at the final time point. *X_ij_* can be directly computed from the amounts of biomass for different species at the end of the COMETS simulations. If *B_ij_* is the final amount of biomass for organism *i* in coculture with organism *j*, and the diagonal element *B_ii_* is the biomass of *i* in monoculture, then the relative yield is defined as *X_ij_* = (*B_ij_* − *B_ii_*)/*B_ii_*, where an *X_ij_* of <0 indicates strain *i* (i.e., the responder) is detrimentally affected by its partner. Correspondingly, an *X_ij_* of 0 indicates no effect and an *X_ij_* of >0 indicates a positive effect of *j* on *i*.

For this case study, the feature profile *F*^*(i)*^ for species *i* encodes the presence (1) or absence (0) of each of 194 possible exchange reactions (corresponding to the columns in the *S* matrix). It is important to note that these feature vectors are equivalent to functional annotations based on genomes, e.g., the profiles of the presence/absence of specific genes. They do not depend on the fluxes that can be eventually computed for each of the corresponding reactions.

In addition to implementing the random forest algorithm, as described below in “Implementation of random forest,” a simple classifier was built on the basis of the Jaccard distance (JD) between two feature vectors *F*^(*i*)^ and *F*^(*j*)^, defined as

$$\text{JD}\left(F^{\left(i\right)},F^{\left(j\right)}\right)=1-\left(F^{\left(i\right)}\cap F^{\left(j\right)}\right)/\left(F^{\left(i\right)}\cup F^{\left(j\right)}\right).$$

### Data for case study of auxotrophic E. coli.

The measured growth responses of individual E. coli strains and biosynthetic costs of amino acids were obtained from the supplemental files provided in reference 36. In that study, 14 strains of amino acid auxotrophic E. coli were generated by knocking out single genes. The cocultures were reported as being inoculated in 200 μl of M9 glucose medium in 96-well microtiter plates at an initial cell density of 10^7^ cells/ml and incubated at 30°C for 84 h, at which point the fold change in growth relative to the initial inoculum for each strain was determined by plating, counting colonies, and quantitative PCR (qPCR) to identify strain proportions. In this case, the feature vector *F*^(*i*)^ (length *n =* 14) encodes the presence/absence of biosynthetic capabilities for each of the 14 amino acids, and the coculture phenotype *X_ij_* corresponds to the fold change of strain *i* in coculture with strain *j* at the final time point, which may represent the final growth yield. On the basis of the original data set, batch effects (e.g., evaporation) or mutations did not affect the quantitative estimate of the reported yield and thus the outcome of our analysis. However, a further scrutiny of the level of precision in yield measurements and corresponding estimates of how experimental errors might affect machine learning outcomes would be an important subject for future follow-up studies.

### Data for case study of soil community.

The results of an experimental study of 20 soil microbial strains in which all pairwise coculture experiments were performed in a yeast extract nutrient broth medium were obtained (3). The survival of different strains after 5 dilution cycles was estimated by plating coculture medium and counting colonies and was verified with next-generation sequencing. For our analysis, *X_ij_* encoded the reported persistence (*X_ij_* = 1) or exclusion (*X_ij_* = 0) of strain *i* when cocultured with strain *j*. To generate feature vectors *F*^(*i*)^ for this community, the 16s rRNA sequence of each strain was downloaded from GenBank (47), and PICRUSt (38) was used to predict the presence of KEGG modules. The KEGG modules for 18 strains were obtained, and each strain was represented by a binary trait vector of 79 modules (see Table S4 in the supplemental material).

### Implementation of random forest.

We used the randomForest R library (26). Random forests are ensemble classifiers that aggregate the results of many individual decision trees. This specific algorithm utilizes two hyperparameters: the number of training trees (nTree) and the number of predictors to consider at each split point (mTry). The default settings of nTree and mTry were near optimal for our *in silico* data set (Fig. S4); therefore, only the default settings were used for the remainder of the study. Each tree in the random forest was assigned a synthetic data set that is of the same size as the training set but generated through sampling with replacement. The average tree was thus trained on approximately two-thirds of the observations; these observations are referred to as in-bag samples. The remaining one-third of the observations not in the synthetic data sets are referred to as out-of-bag samples. The new synthetic data set was placed at the root node of a new tree; next, a randomly selected subset of predictive features was queried for the best split of the data into two child nodes. This process was repeated at each node until a stop criterion was met. The classification accuracy of individual trees was assessed by using them to predict their out-of-bag samples and recording the results. The random forest then makes a classification call for individual samples according to the class predicted by the majority of the trees. The accuracy was evaluated on the full training set with out-of-bag performance metrics and has been shown to be equivalent to 5-fold cross-validation (26). The ratio of the votes of the out-of-bag trees was used to construct ROC curves (see “ROC curves” below). See reference 19 for a full description of the algorithm.

10.1128/mSystems.00181-18.3 FIG S3 For 3 subset sizes of the *in silico* dataset (100, 500, and 1,000), the respective number of samples was randomly selected to use for training a random forest. The AUCs of the ROC curves were calculated using the Jaccard distance as a single threshold on the training set, the vote proportions on the out-of-bag samples from the random forest on the training set, and the vote proportions of the random forest on the samples in the test set. This process was repeated 5 times for each subset. Download FIG S3, PDF file, 0.4 MB. Copyright © 2018 DiMucci et al. 2018 DiMucci et al. This content is distributed under the terms of the Creative Commons Attribution 4.0 International license.

10.1128/mSystems.00181-18.4 FIG S4 (A) The out-of-bag error rate as a function of nTree for the random forest model trained on the full *in silico* data. Error converges at ∼150 trees; the green line is the error rate for nonnegative responses, the red line is the error rate for negative responses. (B) A random subset of 500 samples of the *in silico* data and for the series of possible values of mTry (1, 2, 4, 6, 76, 95, 114, 133, 152, 171, 190, 209, 228, 247, 266, 285, 304, 323, 342, 361, and 380) was selected to see if tuning the hyperparameter mTry resulted in benefits to performance. The AUCs were calculated for the ROC curves obtained from the subsequent class votes. This process was done for 5 random subsets of 500 samples. Points that are the same color in the figure correspond to AUC results from the same series. Download FIG S4, PDF file, 0.05 MB. Copyright © 2018 DiMucci et al. 2018 DiMucci et al. This content is distributed under the terms of the Creative Commons Attribution 4.0 International license.

10.1128/mSystems.00181-18.5 FIG S5 Phylogenetic classifications for each metabolic model were used as an alternative set of features. The out-of-bag error for a model trained on the full 9,900 sample data set is ∼21.18% (black line); the green line is the error rate for nonnegative responses and red line is the error rate for negative responses. Download FIG S5, PDF file, 0.01 MB. Copyright © 2018 DiMucci et al. 2018 DiMucci et al. This content is distributed under the terms of the Creative Commons Attribution 4.0 International license.

### Balanced accuracy.

The balanced accuracy for evaluating the performance of classifiers on independent test sets and on the OOB samples when the model was trained using the full data set is reported. This metric is based on the values from the confusion matrix: true positive (TP), true negative (TN), false positive (FP), and false negative (FN). The balanced accuracy is calculated as [TP/(TP + FN) + TN/(TN + FP)]/2.

### ROC curve.

To evaluate the random forest classifiers for each case study, the receiver operator curve (ROC) from the model trained on the full set of available data was determined. Using the out-of-bag voting proportions, the true-positive rate (sensitivity) was plotted against the false-positive rate (1 − specificity) as the classification threshold was increased from the minimum value to the maximal value. In the context of the random forest algorithm, the classification threshold is the fraction of out-of-bag votes for the positive class. After generating the ROC curve, the area under the curve (AUC) as calculated with the “AUC” package in R (48).

### Learning curves.

To construct the learning curves, a set of fractions was defined, *r* = [0.05, 0.1, 0.2, 0.3, 0.4, 0.5, 0.6, 0.7, 0.8, 0.9, 0.95], for evaluating the balanced accuracy of the model using cross-validation. For all cross-validation experiments, observations *X_ij_* and *X_ji_* were both either in the training set or in the test set. For each fraction in *r*, a subset of the community matrix of the corresponding size was randomly selected to use as a training set and the remaining data were reserved as an independent test set. This process was repeated until at least 10 subsets of training data were selected for each value in *r*. The median balanced accuracy of classifiers was then calculated for each fraction. To investigate the effect of the community size on the learning curve, a set of community sizes was defined (*c* = [10, 20, 30, 40, 50, 60, 70, 80, 90]). For each community size in *c*, five community submatrices were randomly selected from the full *in silico* community matrix. Then, the learning curve was determined for each subcommunity. For each size in *c*, the median learning curve for balanced accuracy of each community size was calculated and is reported in Fig. 2D.

### Variable importance plots.

Variable importance plots are commonly used with random forests to evaluate which variables are the most important for the model by comparing their mean decrease in accuracy scores. The mean decrease in accuracy is a measurement of the change in the accuracy of the forest’s predictions when the variable in question is randomly permuted (20). Here, it was used for the relative ranking of the global importance of each feature. The randomForest package automatically generates the variable importance plots, which are shown in Fig. 2E, 4E, and 5C.

### Feature contributions for binary classifications.

The calculation of feature contributions was described previously (22). This calculation quantifies the effect of a given variable on the classification of a specific sample *j*. After the training of a random forest with *T* trees, the number of training samples at node *k* that belong to each of the two classes (C_1_ and C_2_) can be counted for each tree *t* and node *k* in the path followed by sample *j* in tree *t*. The fraction of samples belonging to C_1_ is indicated by *Y*_*t,k*_^*j*^. The following steps are performed to evaluate the contribution of an individual feature *f* in classifying a specific sample. (i) At each node where feature *f* is the splitting variable, the local increment (*L*_*t,k,f*_^*j*^) in the fraction of samples belonging to class C_1_ is calculated as $L_{t,k,f}^{j}=Y_{t,k+1}^{j}-\;Y_{t,k}^{j}$. (ii) The mean sample-specific contribution of a given feature *f* across all trees is obtained by averaging over all the local increments, i.e.,

$$\phi_{f}^{j}=\frac{\sum_{t=1}^{T}L_{t,k,f}^{j}}{T}$$

Feature contributions for all case studies were computed on out-of-bag trees using the forestFloor package available in R (49).

### Data availability.

The code and data tables necessary to reproduce all of our figures and analyses are hosted at https://github.com/ddimucci/MicrobialCommunities.
