# Supplementary figures and images for: Machine Learning Reveals Missing Edges and Putative Interaction Mechanisms in Microbial Ecosystem Networks
Source: mSystems. 2018 Oct 30;3(5):e00181-18. doi: 10.1128/mSystems.00181-18 (PMC6208640; doi:10.1128/mSystems.00181-18)

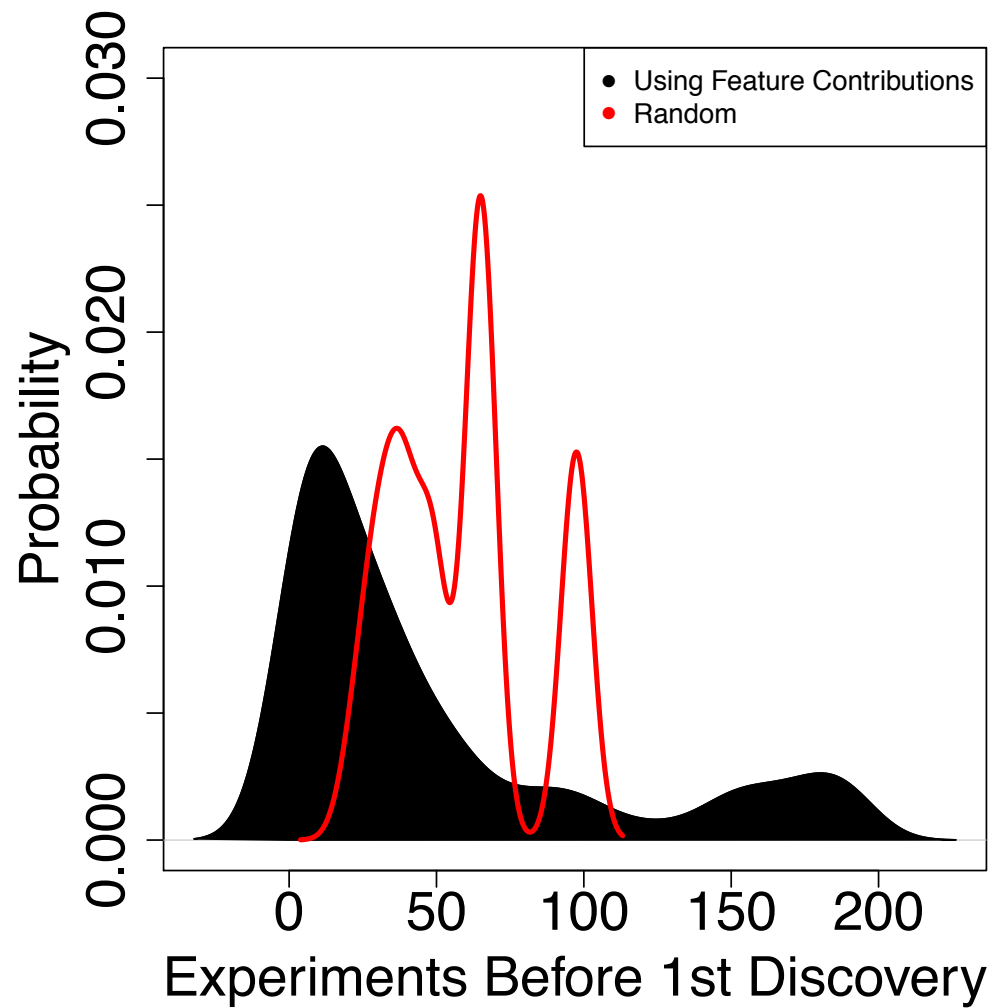

Supplement: FIG S1 [file sys005182279sf1.pdf]

A

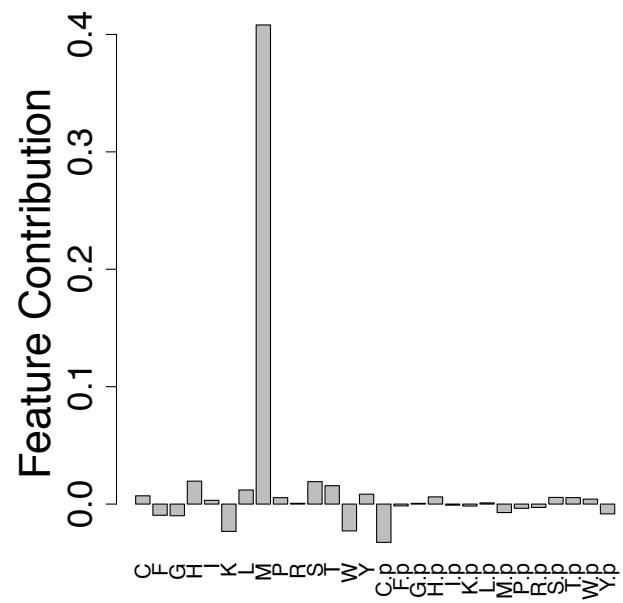

B

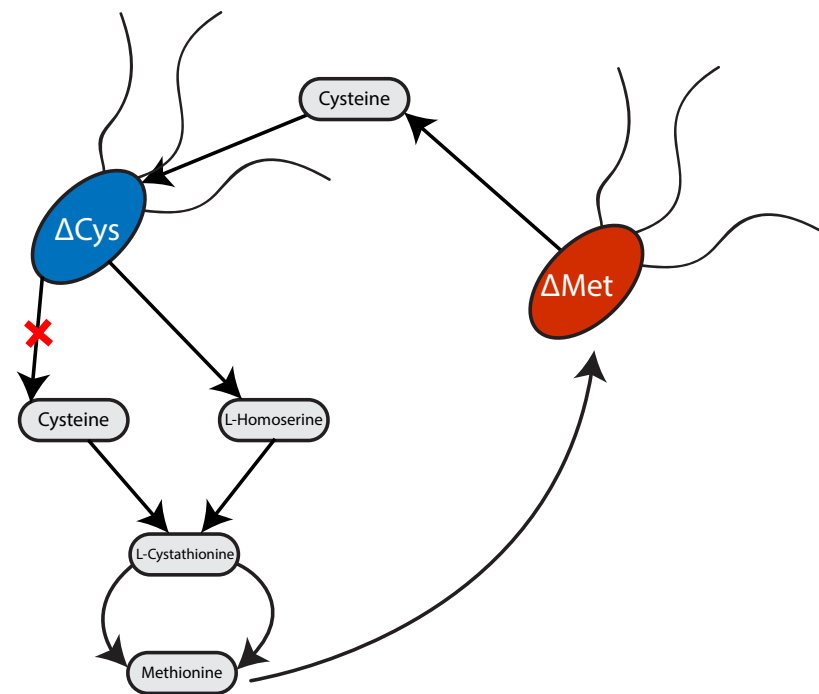

Supplement: FIG S2 [file sys005182279sf2.pdf]

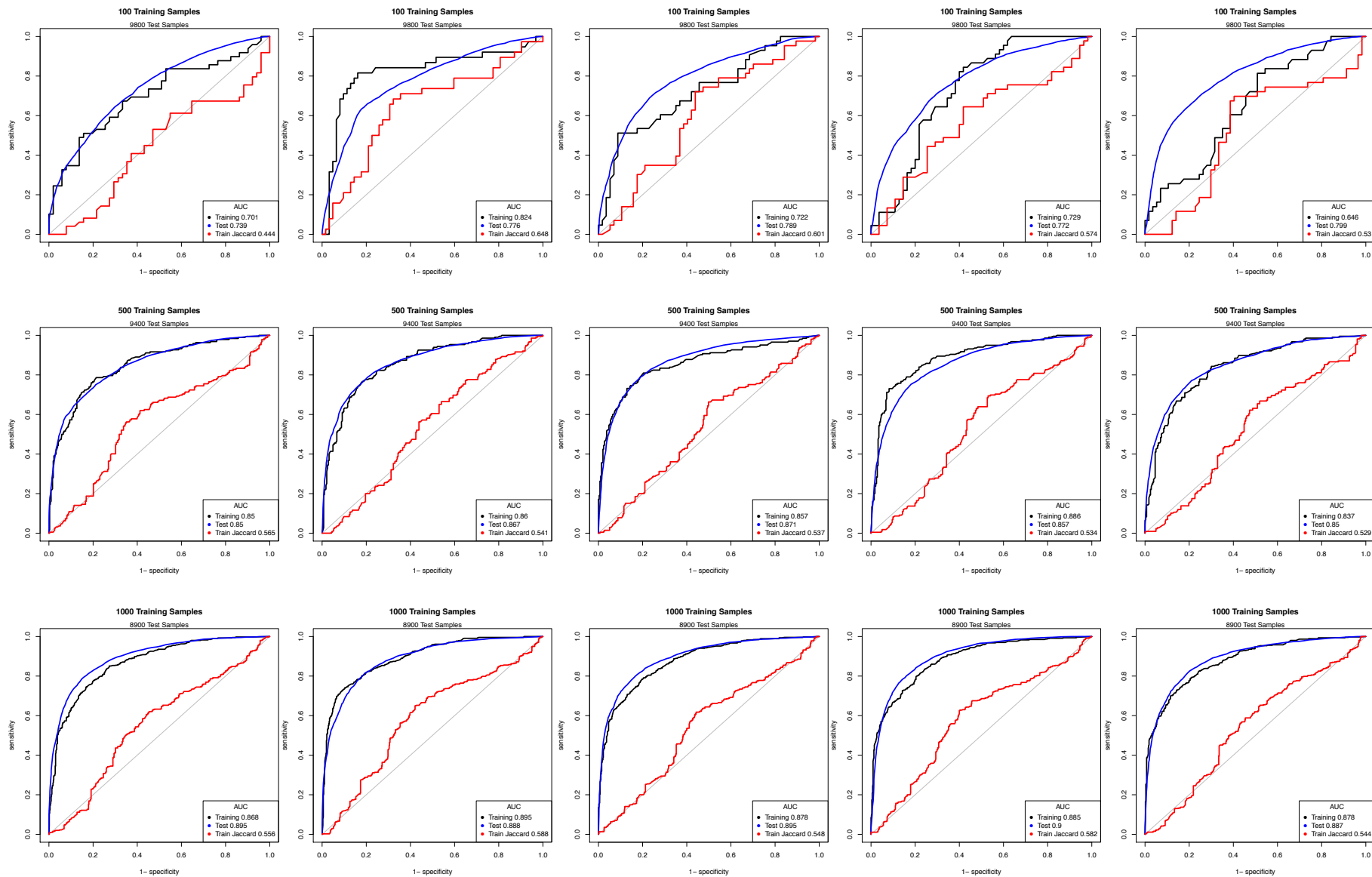

Supplement: FIG S3 [file sys005182279sf3.pdf]

A

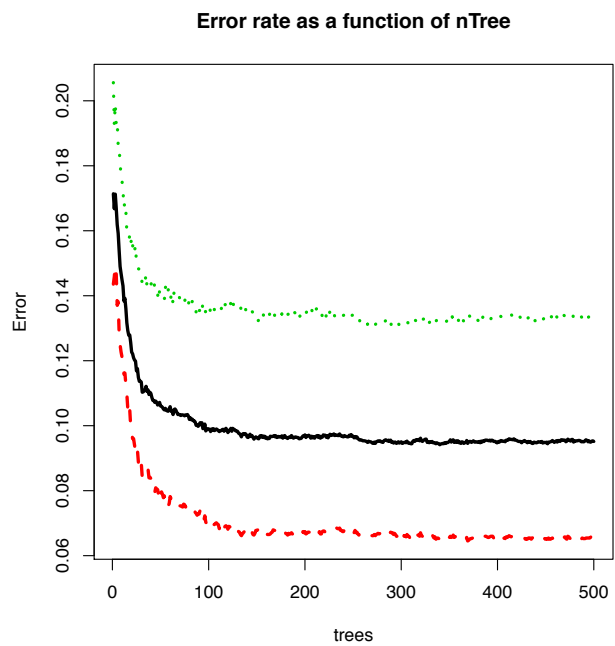

B

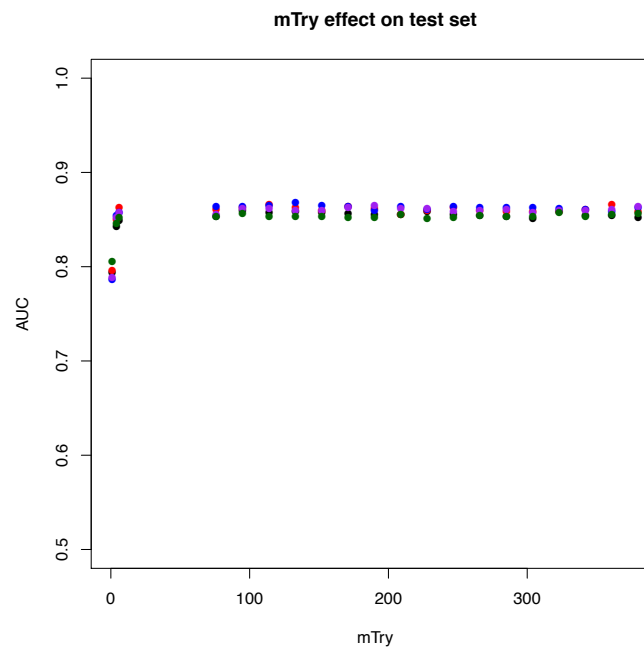

Supplement: FIG S4 [file sys005182279sf4.pdf]

Error rate as a function of nTree

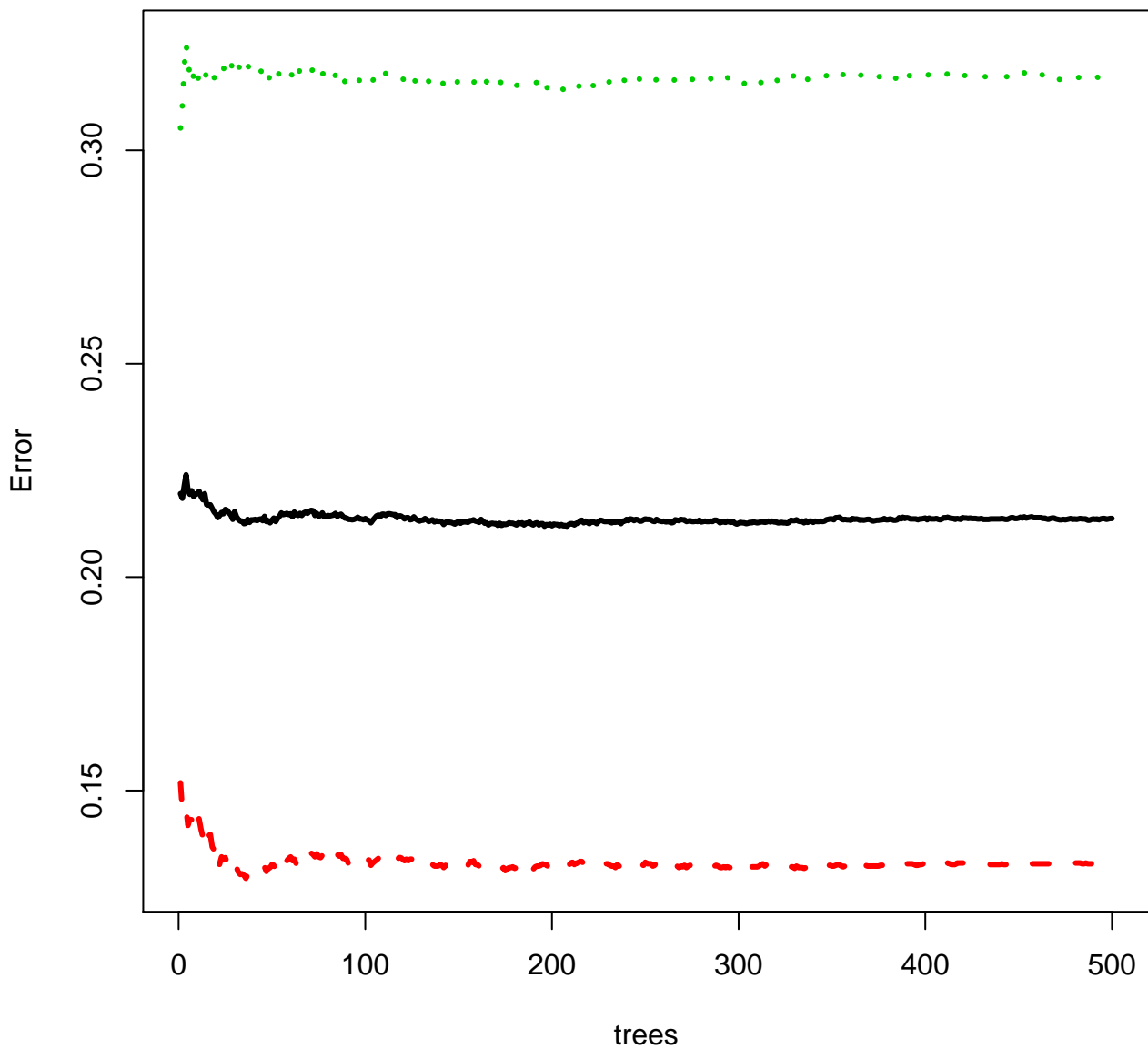

Supplement: FIG S5 [file sys005182279sf5.pdf]
