# Supplementary material for: Machine Learning Reveals Missing Edges and Putative Interaction Mechanisms in Microbial Ecosystem Networks
Source: mSystems. 2018 Oct 30;3(5):e00181-18. doi: 10.1128/mSystems.00181-18 (PMC6208640; doi:10.1128/mSystems.00181-18)
Supplement: TABLE S1 [file sys005182279st1.pdf]

Name	Times Found Firs	Median Rank
D-Fructose	1041	4
gly-asn-L	347	9
ala-L-asp-L	308	8
gly-asp-L	261	8
L-Glutamine	252	3
L-Lysine	245	2
Deoxyguanosine	197	2
Inosine	192	3
G3P	176	2
Deoxycytodine	161	3
Maltose	151	6
Aminoethanol	149	2
D-Ribose	145	2
Formate	142	2
L-Proline	116	6.5
D-Glucose	113	5
Spermidine	99	5
Deoxyinosine	98	3
L-alanglycine	86	6
Riboflavin	82	5
L-Glutamate	80	4
meso-2,6-Diamir	75	1
Adenosine	66	3
Cytidine	66	4
L-Valine	59	4
N-Acetyl-D-gluc	58	3
Fumarate	58	1
Cys-Gly	51	3
Gly-Tyr	47	11
Gly-Phe	47	12
L-Isoleucine	44	5
ocdca	33	2
L-Arginine	32	2
ala-L-glu-L	29	9
gly-pro-L	28	11.5
Deoxyadenosine	26	6
L-Lactate	24	1

Gly-Leu	23	10
Uracil	20	2.5
PAN	20	6
Galactose	19	3
Guanosine	18	1
Uridine	17	2
L-Alanine	17	2
Putrescine	17	3
Gly-Met	16	2
L-Serine	15	4
TRHL	14	2
XAN	13	6
Ala-Leu	13	8
Thymidine	13	4
ala-L-Thr-L	11	9
NH3	11	7
Citrate	10	7.5
Succinate	10	1
Ornithine	9	12
D-Alanine	8	4
Ala-His	8	9.5
Menaquinone 7	8	4.5
L-Methionine	6	6.5
Folate	6	3
LACT	5	3
HYXN	5	10
Nicotinamide rib	5	8
Nicotinamide	5	3
Heme	5	1
Thiamin	5	3
L-Tryptophan	4	7
Sucrose	3	4
L-Leucine	3	7
H2S2O3	2	2.5
CELB	2	14.5
L-Phenylalanine	2	2.5
Dephospho-CoA	2	15.5
GLUM	2	5.5

L-Threonine	1	1
Isoprene	1	2
met-L-ala-L	1	98
4-Hydroxybenzo	1	3
D-Arabinose	1	2
2-Oxoglutarate	1	1
L-Malate	1	12
