# Supplementary material for: Machine Learning Reveals Missing Edges and Putative Interaction Mechanisms in Microbial Ecosystem Networks
Source: mSystems. 2018 Oct 30;3(5):e00181-18. doi: 10.1128/mSystems.00181-18 (PMC6208640; doi:10.1128/mSystems.00181-18)
Supplement: TABLE S2 [file sys005182279st2.pdf]

Model Seed ID	Times Competed For
cpd15605	3205
cpd15606	3153
cpd01017	3057
cpd11584	2842
cpd11581	2836
cpd00322	2745
cpd11589	2723
cpd00027	2651
cpd00156	2521
cpd11593	2482
cpd00220	2399
cpd11582	2211
cpd00039	1881
cpd00226	1876
cpd00082	1875
cpd00129	1849
cpd00179	1740
cpd11586	1642
cpd11590	1595
cpd00644	1471
cpd00065	1145
cpd11588	1141
cpd00023	1028
cpd00080	931
cpd11583	918
cpd00092	886
cpd00013	871
cpd00107	856
cpd00105	855
cpd00051	835
cpd00367	827
cpd00118	824
cpd00654	800
cpd00053	790
cpd00122	757
cpd15604	729
cpd00182	715
cpd00054	691
cpd00246	685

cpd11585	634
cpd00249	568
cpd00355	560
cpd00794	528
cpd00516	518
cpd03279	489
cpd11591	485
cpd00264	461
cpd00438	443
cpd00277	426
cpd00305	392
cpd00159	389
cpd00106	363
cpd00393	363
cpd00036	339
cpd00117	339
cpd00064	337
cpd00137	325
cpd00276	320
cpd00047	319
cpd00311	296
cpd00108	280
cpd00309	280
cpd00033	279
cpd00162	268
cpd00028	253
cpd11606	201
cpd00069	188
cpd00655	163
cpd00060	162
cpd00184	145
cpd00130	125
cpd00041	121
cpd00035	104
cpd00066	101
cpd01080	82
cpd00307	58
cpd03847	57
cpd00208	55
cpd03198	50

cpd00550	46
cpd00136	30
cpd00133	27
cpd00161	22
cpd00218	22
cpd00158	20
cpd00224	12
cpd00492	12
cpd00268	11
cpd00100	10
cpd00359	10
cpd01217	6
cpd01914	6
cpd16336	6
cpd00132	5
cpd00215	5
cpd00075	4
cpd00076	4
cpd00084	4
cpd00142	4
cpd00176	4
cpd03422	4
cpd08636	4
cpd00239	3
cpd00024	2
cpd00185	2
cpd00793	2
cpd01741	2
cpd00119	1
cpd00314	1
cpd00006	0
cpd00012	0
cpd00079	0
cpd00098	0
cpd00104	0
cpd00121	0
cpd00138	0
cpd00139	0
cpd00154	0
cpd00164	0

cpd00209	0
cpd00210	0
cpd00211	0
cpd00214	0
cpd00216	0
cpd00221	0
cpd00222	0
cpd00232	0
cpd00235	0
cpd00244	0
cpd00266	0
cpd00280	0
cpd00281	0
cpd00298	0
cpd00308	0
cpd00338	0
cpd00357	0
cpd00395	0
cpd00396	0
cpd00412	0
cpd00423	0
cpd00441	0
cpd00531	0
cpd00540	0
cpd00573	0
cpd00588	0
cpd00609	0
cpd00635	0
cpd00637	0
cpd00652	0
cpd00653	0
cpd00681	0
cpd00797	0
cpd00811	0
cpd00870	0
cpd00971	0
cpd01012	0
cpd01015	0
cpd01030	0
cpd01048	0

cpd01092	0
cpd01155	0
cpd01171	0
cpd01242	0
cpd01262	0
cpd01329	0
cpd01912	0
cpd02227	0
cpd03048	0
cpd03343	0
cpd03424	0
cpd03696	0
cpd03724	0
cpd03725	0
cpd04097	0
cpd04098	0
cpd08023	0
cpd08305	0
cpd08306	0
cpd09878	0
cpd11574	0
cpd11575	0
cpd11576	0
cpd11578	0
cpd11579	0
cpd11580	0
cpd11587	0
cpd11592	0
cpd11595	0
cpd11596	0
cpd11597	0
cpd15269	0
cpd15302	0
cpd15603	0
cpd16062	0
