# Supplementary material for: Machine Learning Reveals Missing Edges and Putative Interaction Mechanisms in Microbial Ecosystem Networks
Source: mSystems. 2018 Oct 30;3(5):e00181-18. doi: 10.1128/mSystems.00181-18 (PMC6208640; doi:10.1128/mSystems.00181-18)
Supplement: TABLE S3 [file sys005182279st3.pdf]

**Predictive Rank of Knocked Out (KO) Amino Acids**	**Total Occurrences (Fraction of samples)**
Receiver KO 1st	140 (.769)
Giver KO 1st	40 (.22)
Receiver KO 2nd	35 (.192)
Giver KO 2nd	97 (.533)
Receiver KO 1st/Giver KO 2nd	97 (.533)
Giver KO 1st/Receiver KO 2nd	35 (.192)
