# Supplementary material for: Machine Learning Reveals Missing Edges and Putative Interaction Mechanisms in Microbial Ecosystem Networks
Source: mSystems. 2018 Oct 30;3(5):e00181-18. doi: 10.1128/mSystems.00181-18 (PMC6208640; doi:10.1128/mSystems.00181-18)
Supplement: TABLE S4 [file sys005182279st4.pdf]

Module.Name	Module.ID
Sulfate transport system	M00185
Tungstate transport system	M00186
NitT/TauT family transport system	M00188
Molybdate transport system	M00189
Iron(III) transport system	M00190
Putative thiamine transport system	M00192
Putative spermidine/putrescine transport system	M00193
Maltose/maltodextrin transport system	M00194
Raffinose/stachyose/melibiose transport system	M00196
Putative fructooligosaccharide transport system	M00197
Putative sn-glycerol-phosphate transport system	M00198
L-Arabinose/lactose transport system	M00199
Putative sorbitol/mannitol transport system	M00200
alpha-Glucoside transport system	M00201
N-Acetylglucosamine transport system	M00205
Cellobiose transport system	M00206
Putative multiple sugar transport system	M00207
Glycine betaine/proline transport system	M00208
Osmoprotectant transport system	M00209
Phospholipid transport system	M00210
Putative ABC transport system	M00211
Ribose transport system	M00212
D-Xylose transport system	M00215
Multiple sugar transport system	M00216
AI-2 transport system	M00219
Putative simple sugar transport system	M00221
Phosphate transport system	M00222
Phosphonate transport system	M00223
Putative glutamine transport system	M00228
Glutamate/aspartate transport system	M00230
Glutamate transport system	M00233
Putative polar amino acid transport system	M00236
Branched-chain amino acid transport system	M00237
D-Methionine transport system	M00238
Peptides/nickel transport system	M00239
Iron complex transport system	M00240
Zinc transport system	M00242
Manganese/iron transport system	M00243
Putative zinc/manganese transport system	M00244

Cobalt/nickel transport system	M00245
Nickel transport system	M00246
Putative ABC transport system	M00247
Lipopolysaccharide transport system	M00250
Teichoic acid transport system	M00251
Lipooligosaccharide transport system	M00252
Sodium transport system	M00253
ABC-2 type transport system	M00254
Cell division transport system	M00256
Hemin transport system	M00257
Putative ABC transport system	M00258
Heme transport system	M00259
Spermidine/putrescine transport system	M00299
Bacitracin transport system	M00314
Uncharacterized ABC transport system	M00315
Manganese/zinc/iron transport system	M00319
alpha-Hemolysin/cyclolysin transport system	M00325
RTX toxin transport system	M00326
Glutathione transport system	M00348
Microcin C transport system	M00349
Competence-related DNA transformation transporter	M00429
KdpD-KdpE (potassium transport) two-component regula	M00454
TctE-TctD (tricarboxylic acid transport) two-component	M00457
BceS-BceR (bacitracin transport) two-component regulat	M00469
CitS-CitT (magnesium-citrate transport) two-component	M00487
MalK-MalR (malate transport) two-component regulatory	M00490
arabinogalactan oligomer/maltooligosaccharide transport	M00491
DctB-DctD (C4-dicarboxylate transport) two-component	M00504
AlgE-type Mannuronan C-5-Epimerase transport system	M00571
Biotin transport system	M00581
Energy-coupling factor transport system	M00582
Arabinosaccharide transport system	M00602
Glucose/mannose transport system	M00605
N,N'-Diacetylchitobiose transport system	M00606
Tetracycline resistance, TetA transporter	M00668
gamma-Hexachlorocyclohexane transport system	M00669
Mce transport system	M00670
Macrolide resistance, MacAB-TolC transporter	M00709
Bacitracin resistance, VraDE transporter	M00737
Bacitracin resistance, BceAB transporter	M00738
