# Supplementary material for: Machine Learning Reveals Missing Edges and Putative Interaction Mechanisms in Microbial Ecosystem Networks
Source: mSystems. 2018 Oct 30;3(5):e00181-18. doi: 10.1128/mSystems.00181-18 (PMC6208640; doi:10.1128/mSystems.00181-18)
Supplement: TABLE S5 [file sys005182279st5.pdf]

Responder	Partner	top Module	Contribution
Bacillus toyonensis 1	Bacillus toyonensis 2	GraS-GraR (cationic antimicrobial peptide transport) two-component regulatory system	Survival
Bacillus toyonensis 1	Cupriavidus basilensis	gamma-Hexachlorocyclohexane transport system	Survival
Bacillus toyonensis 1	Bacillus amyloliquefaciens 1	GraS-GraR (cationic antimicrobial peptide transport) two-component regulatory system	Survival
Bacillus toyonensis 1	Bacillus safensis	GraS-GraR (cationic antimicrobial peptide transport) two-component regulatory system	Survival
Bacillus toyonensis 1	Bacillus amyloliquefaciens 2	gamma-Hexachlorocyclohexane transport system	Survival
Bacillus toyonensis 1	Bacillus altitudinis	GraS-GraR (cationic antimicrobial peptide transport) two-component regulatory system	Survival
Bacillus toyonensis 1	Bacillus flexus	gamma-Hexachlorocyclohexane transport system	Survival
Bacillus toyonensis 1	Bacillus aryabhattai 1	gamma-Hexachlorocyclohexane transport system	Survival

Bacillus toyonensis 1	Arthrobacter humicola	gamma-Hexachlorocyclohexane transport system	Survival
Bacillus toyonensis 1	Arthrobacter nitroguajacolicus	gamma-Hexachlorocyclohexane transport system	Survival
Bacillus toyonensis 1	Bacillus aryabhattai 2	gamma-Hexachlorocyclohexane transport system	Survival
Bacillus toyonensis 1	Rummeliibacillus pycnus	gamma-Hexachlorocyclohexane transport system	Survival
Bacillus toyonensis 1	Bacillus aryabhattai 3	gamma-Hexachlorocyclohexane transport system	Survival
Bacillus toyonensis 1	Arthrobacter oxydans	gamma-Hexachlorocyclohexane transport system	Survival
Bacillus toyonensis 1	Bacillus simplex 2	gamma-Hexachlorocyclohexane transport system	Survival
Bacillus toyonensis 1	Bacillus simplex 1	gamma-Hexachlorocyclohexane transport system	Survival
Bacillus toyonensis 2	Bacillus toyonensis 1	Glutamate transport system	Survival
Bacillus toyonensis 2	Bacillus toyonensis 3	Glutamate transport system	Survival
Bacillus toyonensis 2	Cupriavidus basilensis	gamma-Hexachlorocyclohexane transport system	Survival

Bacillus toyonensis 2	Bacillus amyloliquefaciens 1	Glutamate transport system	Extinction
Bacillus toyonensis 2	Bacillus safensis	Glutamate transport system	Survival
Bacillus toyonensis 2	Bacillus amyloliquefaciens 2	gamma-Hexachlorocyclohexane transport system	Survival
Bacillus toyonensis 2	Bacillus altitudinis	Glutamate transport system	Survival
Bacillus toyonensis 2	Bacillus flexus	gamma-Hexachlorocyclohexane transport system	Survival
Bacillus toyonensis 2	Bacillus aryabhattai 1	gamma-Hexachlorocyclohexane transport system	Survival
Bacillus toyonensis 2	Arthrobacter humicola	gamma-Hexachlorocyclohexane transport system	Survival
Bacillus toyonensis 2	Arthrobacter nitroguajacolicus	gamma-Hexachlorocyclohexane transport system	Survival
Bacillus toyonensis 2	Bacillus aryabhattai 2	gamma-Hexachlorocyclohexane transport system	Survival
Bacillus toyonensis 2	Rummeliibacillus pycnus	gamma-Hexachlorocyclohexane transport system	Survival
Bacillus toyonensis 2	Bacillus aryabhattai 3	gamma-Hexachlorocyclohexane transport system	Survival

Bacillus toyonensis 2	Arthrobacter oxydans	gamma-Hexachlorocyclohexane transport system	Survival
Bacillus toyonensis 2	Bacillus simplex 2	gamma-Hexachlorocyclohexane transport system	Survival
Bacillus toyonensis 2	Bacillus simplex 1	gamma-Hexachlorocyclohexane transport system	Survival
Bacillus toyonensis 3	Bacillus toyonensis 2	GraS-GraR (cationic antimicrobial peptide transport) two-component regulatory system	Survival
Bacillus toyonensis 3	Cupriavidus basilensis	gamma-Hexachlorocyclohexane transport system	Extinction
Bacillus toyonensis 3	Bacillus amyloliquefaciens 1	GraS-GraR (cationic antimicrobial peptide transport) two-component regulatory system	Survival
Bacillus toyonensis 3	Bacillus safensis	GraS-GraR (cationic antimicrobial peptide transport) two-component regulatory system	Survival
Bacillus toyonensis 3	Bacillus amyloliquefaciens 2	gamma-Hexachlorocyclohexane transport system	Extinction
Bacillus toyonensis 3	Bacillus altitudinis	GraS-GraR (cationic antimicrobial peptide transport) two-component regulatory system	Survival

Bacillus toyonensis 3	Bacillus flexus	gamma-Hexachlorocyclohexane transport system	Survival
Bacillus toyonensis 3	Bacillus aryabhattai 1	gamma-Hexachlorocyclohexane transport system	Survival
Bacillus toyonensis 3	Arthrobacter humicola	gamma-Hexachlorocyclohexane transport system	Survival
Bacillus toyonensis 3	Arthrobacter nitroguajacolicus	gamma-Hexachlorocyclohexane transport system	Survival
Bacillus toyonensis 3	Bacillus aryabhattai 2	gamma-Hexachlorocyclohexane transport system	Survival
Bacillus toyonensis 3	Rummeliibacillus pycnus	gamma-Hexachlorocyclohexane transport system	Survival
Bacillus toyonensis 3	Bacillus aryabhattai 3	gamma-Hexachlorocyclohexane transport system	Survival
Bacillus toyonensis 3	Arthrobacter oxydans	gamma-Hexachlorocyclohexane transport system	Survival
Bacillus toyonensis 3	Bacillus simplex 2	gamma-Hexachlorocyclohexane transport system	Survival
Bacillus toyonensis 3	Bacillus simplex 1	gamma-Hexachlorocyclohexane transport system	Survival

Cupriavidus basilensis	Bacillus toyonensis 1	gamma-Hexachlorocyclohexane transport system	Survival
Cupriavidus basilensis	Bacillus toyonensis 2	gamma-Hexachlorocyclohexane transport system	Survival
Cupriavidus basilensis	Bacillus toyonensis 3	gamma-Hexachlorocyclohexane transport system	Survival
Cupriavidus basilensis	Bacillus amyloliquefaciens 1	gamma-Hexachlorocyclohexane transport system	Extinction
Cupriavidus basilensis	Bacillus safensis	gamma-Hexachlorocyclohexane transport system	Survival
Cupriavidus basilensis	Bacillus amyloliquefaciens 2	Glutamate transport system	Survival
Cupriavidus basilensis	Bacillus altitudinis	gamma-Hexachlorocyclohexane transport system	Survival
Cupriavidus basilensis	Bacillus flexus	Glutamate transport system	Survival
Cupriavidus basilensis	Bacillus aryabhattai 1	Glutamate transport system	Survival
Cupriavidus basilensis	Arthrobacter humicola	Cationic antimicrobial peptide (CAMP) resistance, VraFG transporter	Survival

Cupriavidus basilensis	Arthrobacter nitroguajacolicus	Cationic antimicrobial peptide (CAMP) resistance, VraFG transporter	Survival
Cupriavidus basilensis	Bacillus aryabhattai 2	Glutamate transport system	Survival
Cupriavidus basilensis	Rummeliibacillus pycnus	Glutamate transport system	Survival
Cupriavidus basilensis	Bacillus aryabhattai 3	Glutamate transport system	Survival
Cupriavidus basilensis	Arthrobacter oxydans	Cationic antimicrobial peptide (CAMP) resistance, VraFG transporter	Survival
Cupriavidus basilensis	Bacillus simplex 2	Cationic antimicrobial peptide (CAMP) resistance, VraFG transporter	Survival
Cupriavidus basilensis	Bacillus simplex 1	Glutamate transport system	Survival
Bacillus amyloliquefaciens 1	Bacillus toyonensis 1	GraS-GraR (cationic antimicrobial peptide transport) two-component regulatory system	Extinction
Bacillus amyloliquefaciens 1	Bacillus toyonensis 2	Glutamate/aspartate transport system	Extinction
Bacillus amyloliquefaciens 1	Bacillus toyonensis 3	GraS-GraR (cationic antimicrobial peptide transport) two-component regulatory system	Extinction
Bacillus amyloliquefaciens 1	Cupriavidus basilensis	gamma-Hexachlorocyclohexane transport system	Extinction

Bacillus amyloliquefaciens 1	Bacillus safensis	Glycerol transport system	Extinction
Bacillus amyloliquefaciens 1	Bacillus altitudinis	Iron complex transport system	Survival
Bacillus amyloliquefaciens 1	Bacillus flexus	gamma-Hexachlorocyclohexane transport system	Survival
Bacillus amyloliquefaciens 1	Bacillus aryabhattai 1	gamma-Hexachlorocyclohexane transport system	Survival
Bacillus amyloliquefaciens 1	Arthrobacter humicola	gamma-Hexachlorocyclohexane transport system	Survival
Bacillus amyloliquefaciens 1	Arthrobacter nitroguajacolicus	gamma-Hexachlorocyclohexane transport system	Survival
Bacillus amyloliquefaciens 1	Bacillus aryabhattai 2	gamma-Hexachlorocyclohexane transport system	Survival
Bacillus amyloliquefaciens 1	Rummeliibacillus pycnus	gamma-Hexachlorocyclohexane transport system	Survival
Bacillus amyloliquefaciens 1	Bacillus aryabhattai 3	gamma-Hexachlorocyclohexane transport system	Survival
Bacillus amyloliquefaciens 1	Arthrobacter oxydans	gamma-Hexachlorocyclohexane transport system	Survival

Bacillus amyloliquefaciens 1	Bacillus simplex 2	gamma-Hexachlorocyclohexane transport system	Survival
Bacillus amyloliquefaciens 1	Bacillus simplex 1	gamma-Hexachlorocyclohexane transport system	Survival
Bacillus safensis	Bacillus toyonensis 1	GraS-GraR (cationic antimicrobial peptide transport) two-component regulatory system	Extinction
Bacillus safensis	Bacillus toyonensis 2	Glycine betaine/proline transport system	Extinction
Bacillus safensis	Bacillus toyonensis 3	Cationic antimicrobial peptide transport system	Extinction
Bacillus safensis	Cupriavidus basilensis	gamma-Hexachlorocyclohexane transport system	Survival
Bacillus safensis	Bacillus amyloliquefaciens 1	Glycerol transport system	Extinction
Bacillus safensis	Bacillus amyloliquefaciens 2	gamma-Hexachlorocyclohexane transport system	Extinction
Bacillus safensis	Bacillus altitudinis	gamma-Hexachlorocyclohexane transport system	Survival
Bacillus safensis	Bacillus flexus	gamma-Hexachlorocyclohexane transport system	Survival

Bacillus safensis	Bacillus aryabhattai 1	gamma-Hexachlorocyclohexane transport system	Survival
Bacillus safensis	Arthrobacter humicola	gamma-Hexachlorocyclohexane transport system	Survival
Bacillus safensis	Arthrobacter nitroguajacolicus	gamma-Hexachlorocyclohexane transport system	Survival
Bacillus safensis	Bacillus aryabhattai 2	gamma-Hexachlorocyclohexane transport system	Survival
Bacillus safensis	Rummeliibacillus pycnus	gamma-Hexachlorocyclohexane transport system	Survival
Bacillus safensis	Bacillus aryabhattai 3	gamma-Hexachlorocyclohexane transport system	Survival
Bacillus safensis	Arthrobacter oxydans	gamma-Hexachlorocyclohexane transport system	Survival
Bacillus safensis	Bacillus simplex 2	gamma-Hexachlorocyclohexane transport system	Survival
Bacillus safensis	Bacillus simplex 1	gamma-Hexachlorocyclohexane transport system	Survival
Bacillus amyloliquefaciens 2	Bacillus toyonensis 1	gamma-Hexachlorocyclohexane transport system	Extinction

Bacillus amyloliquefaciens 2	Bacillus toyonensis 2	gamma-Hexachlorocyclohexane transport system	Extinction
Bacillus amyloliquefaciens 2	Bacillus toyonensis 3	gamma-Hexachlorocyclohexane transport system	Extinction
Bacillus amyloliquefaciens 2	Cupriavidus basilensis	Fructose transport system	Survival
Bacillus amyloliquefaciens 2	Bacillus safensis	gamma-Hexachlorocyclohexane transport system	Extinction
Bacillus amyloliquefaciens 2	Bacillus altitudinis	gamma-Hexachlorocyclohexane transport system	Extinction
Bacillus amyloliquefaciens 2	Bacillus flexus	Cationic antimicrobial peptide (CAMP) resistance, VraFG transporter	Survival
Bacillus amyloliquefaciens 2	Bacillus aryabhattai 1	alpha-1,4-Digalacturonate transport system	Survival
Bacillus amyloliquefaciens 2	Arthrobacter humicola	Iron complex transport system	Survival
Bacillus amyloliquefaciens 2	Arthrobacter nitroguajacolicus	BceS-BceR (bacitracin transport) two-component regulatory system	Survival
Bacillus amyloliquefaciens 2	Bacillus aryabhattai 2	alpha-1,4-Digalacturonate transport system	Survival

Bacillus amyloliquefaciens 2	Rummeliibacillus pycnus	Cationic antimicrobial peptide (CAMP) resistance, VraFG transporter	Survival
Bacillus amyloliquefaciens 2	Bacillus aryabhattai 3	Cationic antimicrobial peptide (CAMP) resistance, VraFG transporter	Survival
Bacillus amyloliquefaciens 2	Arthrobacter oxydans	Capsular polysaccharide transport system	Survival
Bacillus amyloliquefaciens 2	Bacillus simplex 2	AlgE-type Mannuronan C-5-Epimerase transport system	Survival
Bacillus amyloliquefaciens 2	Bacillus simplex 1	AlgE-type Mannuronan C-5-Epimerase transport system	Survival
Bacillus altitudinis	Bacillus toyonensis 1	GraS-GraR (cationic antimicrobial peptide transport) two-component regulatory system	Extinction
Bacillus altitudinis	Bacillus toyonensis 2	Glutamate/aspartate transport system	Extinction
Bacillus altitudinis	Bacillus toyonensis 3	Cationic antimicrobial peptide transport system	Extinction
Bacillus altitudinis	Cupriavidus basilensis	gamma-Hexachlorocyclohexane transport system	Survival
Bacillus altitudinis	Bacillus amyloliquefaciens 1	D-Allose transport system	Survival

Bacillus altitudinis	Bacillus safensis	gamma-Hexachlorocyclohexane transport system	Extinction
Bacillus altitudinis	Bacillus amyloliquefaciens 2	gamma-Hexachlorocyclohexane transport system	Extinction
Bacillus altitudinis	Bacillus flexus	gamma-Hexachlorocyclohexane transport system	Survival
Bacillus altitudinis	Bacillus aryabhattai 1	gamma-Hexachlorocyclohexane transport system	Survival
Bacillus altitudinis	Arthrobacter humicola	gamma-Hexachlorocyclohexane transport system	Survival
Bacillus altitudinis	Arthrobacter nitroguajacolicus	gamma-Hexachlorocyclohexane transport system	Survival
Bacillus altitudinis	Bacillus aryabhattai 2	gamma-Hexachlorocyclohexane transport system	Survival
Bacillus altitudinis	Rummeliibacillus pycnus	gamma-Hexachlorocyclohexane transport system	Survival
Bacillus altitudinis	Bacillus aryabhattai 3	gamma-Hexachlorocyclohexane transport system	Survival
Bacillus altitudinis	Arthrobacter oxydans	gamma-Hexachlorocyclohexane transport system	Survival

Bacillus altitudinis	Bacillus simplex 2	gamma-Hexachlorocyclohexane transport system	Survival
Bacillus altitudinis	Bacillus simplex 1	gamma-Hexachlorocyclohexane transport system	Survival
Bacillus flexus	Bacillus toyonensis 1	gamma-Hexachlorocyclohexane transport system	Extinction
Bacillus flexus	Bacillus toyonensis 2	gamma-Hexachlorocyclohexane transport system	Extinction
Bacillus flexus	Bacillus toyonensis 3	gamma-Hexachlorocyclohexane transport system	Extinction
Bacillus flexus	Cupriavidus basilensis	alpha-1,4-Digalacturonate transport system	Extinction
Bacillus flexus	Bacillus amyloliquefaciens 1	gamma-Hexachlorocyclohexane transport system	Extinction
Bacillus flexus	Bacillus safensis	gamma-Hexachlorocyclohexane transport system	Extinction
Bacillus flexus	Bacillus amyloliquefaciens 2	Cationic antimicrobial peptide (CAMP) resistance, VraFG transporter	Extinction
Bacillus flexus	Bacillus altitudinis	gamma-Hexachlorocyclohexane transport system	Extinction

Bacillus flexus	Bacillus aryabhattai 1	Cationic antimicrobial peptide (CAMP) resistance, VraFG transporter	Survival
Bacillus flexus	Arthrobacter humicola	Cationic antimicrobial peptide (CAMP) resistance, VraFG transporter	Survival
Bacillus flexus	Arthrobacter nitroguajacolicus	Fluoroquinolone transport system	Extinction
Bacillus flexus	Bacillus aryabhattai 2	Cationic antimicrobial peptide (CAMP) resistance, VraFG transporter	Survival
Bacillus flexus	Rummeliibacillus pycnus	alpha-1,4-Digalacturonate transport system	Survival
Bacillus flexus	Bacillus aryabhattai 3	D-Allose transport system	Survival
Bacillus flexus	Arthrobacter oxydans	Cationic antimicrobial peptide (CAMP) resistance, VraFG transporter	Survival
Bacillus flexus	Bacillus simplex 2	Cationic antimicrobial peptide (CAMP) resistance, VraFG transporter	Survival
Bacillus flexus	Bacillus simplex 1	Cationic antimicrobial peptide (CAMP) resistance, VraFG transporter	Survival

Bacillus aryabhattai 1	Bacillus toyonensis 1	gamma-Hexachlorocyclohexane transport system	Extinction
Bacillus aryabhattai 1	Bacillus toyonensis 2	gamma-Hexachlorocyclohexane transport system	Extinction
Bacillus aryabhattai 1	Bacillus toyonensis 3	gamma-Hexachlorocyclohexane transport system	Extinction
Bacillus aryabhattai 1	Cupriavidus basilensis	alpha-1,4-Digalacturonate transport system	Extinction
Bacillus aryabhattai 1	Bacillus amyloliquefaciens 1	gamma-Hexachlorocyclohexane transport system	Extinction
Bacillus aryabhattai 1	Bacillus safensis	gamma-Hexachlorocyclohexane transport system	Extinction
Bacillus aryabhattai 1	Bacillus amyloliquefaciens 2	Cationic antimicrobial peptide (CAMP) resistance, VraFG transporter	Extinction
Bacillus aryabhattai 1	Bacillus altitudinis	gamma-Hexachlorocyclohexane transport system	Extinction
Bacillus aryabhattai 1	Bacillus flexus	Cationic antimicrobial peptide (CAMP) resistance, VraFG transporter	Extinction

Bacillus aryabhattai 1	Arthrobacter humicola	Cationic antimicrobial peptide (CAMP) resistance, VraFG transporter	Survival
Bacillus aryabhattai 1	Arthrobacter nitroguajacolicus	Cationic antimicrobial peptide (CAMP) resistance, VraFG transporter	Extinction
Bacillus aryabhattai 1	Bacillus aryabhattai 2	Fluoroquinolone transport system	Survival
Bacillus aryabhattai 1	Rummeliibacillus pycnus	alpha-1,4-Digalacturonate transport system	Survival
Bacillus aryabhattai 1	Bacillus aryabhattai 3	alpha-1,4-Digalacturonate transport system	Extinction
Bacillus aryabhattai 1	Arthrobacter oxydans	Cationic antimicrobial peptide (CAMP) resistance, VraFG transporter	Survival
Bacillus aryabhattai 1	Bacillus simplex 2	Cationic antimicrobial peptide (CAMP) resistance, VraFG transporter	Survival
Bacillus aryabhattai 1	Bacillus simplex 1	Cationic antimicrobial peptide (CAMP) resistance, VraFG transporter	Survival
Arthrobacter humicola	Bacillus toyonensis 1	gamma-Hexachlorocyclohexane transport system	Extinction

Arthrobacter humicola	Bacillus toyonensis 2	gamma-Hexachlorocyclohexane transport system	Extinction
Arthrobacter humicola	Bacillus toyonensis 3	gamma-Hexachlorocyclohexane transport system	Extinction
Arthrobacter humicola	Cupriavidus basilensis	Fructose transport system	Survival
Arthrobacter humicola	Bacillus amyloliquefaciens 1	gamma-Hexachlorocyclohexane transport system	Extinction
Arthrobacter humicola	Bacillus safensis	gamma-Hexachlorocyclohexane transport system	Extinction
Arthrobacter humicola	Bacillus amyloliquefaciens 2	Iron complex transport system	Extinction
Arthrobacter humicola	Bacillus altitudinis	gamma-Hexachlorocyclohexane transport system	Extinction
Arthrobacter humicola	Bacillus flexus	Fluoroquinolone transport system	Survival
Arthrobacter humicola	Bacillus aryabhattai 1	Fluoroquinolone transport system	Extinction
Arthrobacter humicola	Arthrobacter nitroguajacolicus	BceS-BceR (bacitracin transport) two-component regulatory system	Survival
Arthrobacter humicola	Bacillus aryabhattai 2	Fluoroquinolone transport system	Survival
Arthrobacter humicola	Rummeliibacillus pycnus	Fluoroquinolone transport system	Extinction
Arthrobacter humicola	Bacillus aryabhattai 3	Fluoroquinolone transport system	Survival

Arthrobacter humicola	Arthrobacter oxydans	Iron complex transport system	Survival
Arthrobacter humicola	Bacillus simplex 2	D-Methionine transport system	Survival
Arthrobacter humicola	Bacillus simplex 1	Fluoroquinolone transport system	Survival
Arthrobacter nitroguajacolicus	Bacillus toyonensis 1	gamma-Hexachlorocyclohexane transport system	Extinction
Arthrobacter nitroguajacolicus	Bacillus toyonensis 2	gamma-Hexachlorocyclohexane transport system	Extinction
Arthrobacter nitroguajacolicus	Bacillus toyonensis 3	gamma-Hexachlorocyclohexane transport system	Extinction
Arthrobacter nitroguajacolicus	Cupriavidus basilensis	Fluoroquinolone transport system	Survival
Arthrobacter nitroguajacolicus	Bacillus amyloliquefaciens 1	gamma-Hexachlorocyclohexane transport system	Extinction
Arthrobacter nitroguajacolicus	Bacillus safensis	gamma-Hexachlorocyclohexane transport system	Extinction
Arthrobacter nitroguajacolicus	Bacillus amyloliquefaciens 2	AlgE-type Mannuronan C-5-Epimerase transport system	Extinction
Arthrobacter nitroguajacolicus	Bacillus altitudinis	gamma-Hexachlorocyclohexane transport system	Extinction
Arthrobacter nitroguajacolicus	Bacillus flexus	Fluoroquinolone transport system	Extinction
Arthrobacter nitroguajacolicus	Bacillus aryabhattai 1	Fluoroquinolone transport system	Extinction

Arthrobacter nitroguajacolicus	Arthrobacter humicola	Branched-chain amino acid transport system	Extinction
Arthrobacter nitroguajacolicus	Bacillus aryabhattai 2	Fluoroquinolone transport system	Survival
Arthrobacter nitroguajacolicus	Rummeliibacillus pycnus	Cationic antimicrobial peptide (CAMP) resistance, VraFG transporter	Extinction
Arthrobacter nitroguajacolicus	Bacillus aryabhattai 3	Fluoroquinolone transport system	Survival
Arthrobacter nitroguajacolicus	Arthrobacter oxydans	Iron complex transport system	Survival
Arthrobacter nitroguajacolicus	Bacillus simplex 2	AlgE-type Mannuronan C-5-Epimerase transport system	Survival
Arthrobacter nitroguajacolicus	Bacillus simplex 1	BceS-BceR (bacitracin transport) two-component regulatory system	Survival
Bacillus aryabhattai 2	Bacillus toyonensis 1	gamma-Hexachlorocyclohexane transport system	Extinction
Bacillus aryabhattai 2	Bacillus toyonensis 2	gamma-Hexachlorocyclohexane transport system	Extinction
Bacillus aryabhattai 2	Bacillus toyonensis 3	gamma-Hexachlorocyclohexane transport system	Extinction
Bacillus aryabhattai 2	Cupriavidus basilensis	Fluoroquinolone transport system	Survival

Bacillus aryabhattai 2	Bacillus amyloliquefaciens 1	gamma-Hexachlorocyclohexane transport system	Extinction
Bacillus aryabhattai 2	Bacillus safensis	gamma-Hexachlorocyclohexane transport system	Extinction
Bacillus aryabhattai 2	Bacillus amyloliquefaciens 2	Cationic antimicrobial peptide (CAMP) resistance, VraFG transporter	Extinction
Bacillus aryabhattai 2	Bacillus altitudinis	gamma-Hexachlorocyclohexane transport system	Extinction
Bacillus aryabhattai 2	Bacillus flexus	gamma-Hexachlorocyclohexane transport system	Extinction
Bacillus aryabhattai 2	Bacillus aryabhattai 1	BceS-BceR (bacitracin transport) two-component regulatory system	Survival
Bacillus aryabhattai 2	Arthrobacter humicola	Cationic antimicrobial peptide (CAMP) resistance, VraFG transporter	Survival
Bacillus aryabhattai 2	Arthrobacter nitroguajacolicus	Fluoroquinolone transport system	Extinction
Bacillus aryabhattai 2	Rummeliibacillus pycnus	alpha-1,4-Digalacturonate transport system	Extinction

Bacillus aryabhattai 2	Bacillus aryabhattai 3	Cationic antimicrobial peptide (CAMP) resistance, VraFG transporter	Extinction
Bacillus aryabhattai 2	Arthrobacter oxydans	Cationic antimicrobial peptide (CAMP) resistance, VraFG transporter	Survival
Bacillus aryabhattai 2	Bacillus simplex 2	Cationic antimicrobial peptide (CAMP) resistance, VraFG transporter	Survival
Bacillus aryabhattai 2	Bacillus simplex 1	Cationic antimicrobial peptide (CAMP) resistance, VraFG transporter	Survival
Rummeliibacillus pycnus	Bacillus toyonensis 1	gamma-Hexachlorocyclohexane transport system	Extinction
Rummeliibacillus pycnus	Bacillus toyonensis 2	gamma-Hexachlorocyclohexane transport system	Extinction
Rummeliibacillus pycnus	Bacillus toyonensis 3	gamma-Hexachlorocyclohexane transport system	Extinction
Rummeliibacillus pycnus	Cupriavidus basilensis	BceS-BceR (bacitracin transport) two-component regulatory system	Extinction

Rummeliibacillus pycnus	Bacillus amyloliquefaciens 1	gamma-Hexachlorocyclohexane transport system	Extinction
Rummeliibacillus pycnus	Bacillus safensis	gamma-Hexachlorocyclohexane transport system	Extinction
Rummeliibacillus pycnus	Bacillus amyloliquefaciens 2	Cationic antimicrobial peptide (CAMP) resistance, VraFG transporter	Extinction
Rummeliibacillus pycnus	Bacillus altitudinis	gamma-Hexachlorocyclohexane transport system	Extinction
Rummeliibacillus pycnus	Bacillus flexus	alpha-1,4-Digalacturonate transport system	Extinction
Rummeliibacillus pycnus	Bacillus aryabhattai 1	alpha-1,4-Digalacturonate transport system	Extinction
Rummeliibacillus pycnus	Arthrobacter humicola	Cationic antimicrobial peptide (CAMP) resistance, VraFG transporter	Survival
Rummeliibacillus pycnus	Arthrobacter nitroguajacolicus	Fluoroquinolone transport system	Survival
Rummeliibacillus pycnus	Bacillus aryabhattai 2	alpha-1,4-Digalacturonate transport system	Extinction
Rummeliibacillus pycnus	Bacillus aryabhattai 3	alpha-1,4-Digalacturonate transport system	Extinction

Rummeliibacillus pycnus	Arthrobacter oxydans	Cationic antimicrobial peptide (CAMP) resistance, VraFG transporter	Survival
Rummeliibacillus pycnus	Bacillus simplex 2	Cationic antimicrobial peptide (CAMP) resistance, VraFG transporter	Survival
Rummeliibacillus pycnus	Bacillus simplex 1	Cationic antimicrobial peptide (CAMP) resistance, VraFG transporter	Survival
Bacillus aryabhattai 3	Bacillus toyonensis 1	gamma-Hexachlorocyclohexane transport system	Extinction
Bacillus aryabhattai 3	Bacillus toyonensis 2	gamma-Hexachlorocyclohexane transport system	Extinction
Bacillus aryabhattai 3	Bacillus toyonensis 3	gamma-Hexachlorocyclohexane transport system	Extinction
Bacillus aryabhattai 3	Cupriavidus basilensis	alpha-1,4-Digalacturonate transport system	Extinction
Bacillus aryabhattai 3	Bacillus amyloliquefaciens 1	gamma-Hexachlorocyclohexane transport system	Extinction
Bacillus aryabhattai 3	Bacillus safensis	gamma-Hexachlorocyclohexane transport system	Extinction

Bacillus aryabhattai 3	Bacillus amyloliquefaciens 2	Cationic antimicrobial peptide (CAMP) resistance, VraFG transporter	Extinction
Bacillus aryabhattai 3	Bacillus altitudinis	gamma-Hexachlorocyclohexane transport system	Extinction
Bacillus aryabhattai 3	Bacillus flexus	Cationic antimicrobial peptide (CAMP) resistance, VraFG transporter	Extinction
Bacillus aryabhattai 3	Bacillus aryabhattai 1	gamma-Hexachlorocyclohexane transport system	Extinction
Bacillus aryabhattai 3	Arthrobacter humicola	Cationic antimicrobial peptide (CAMP) resistance, VraFG transporter	Extinction
Bacillus aryabhattai 3	Arthrobacter nitroguajacolicus	Fluoroquinolone transport system	Extinction
Bacillus aryabhattai 3	Bacillus aryabhattai 2	BceS-BceR (bacitracin transport) two-component regulatory system	Extinction
Bacillus aryabhattai 3	Rummeliibacillus pycnus	alpha-1,4-Digalacturonate transport system	Extinction
Bacillus aryabhattai 3	Arthrobacter oxydans	Cationic antimicrobial peptide (CAMP) resistance, VraFG transporter	Survival

Bacillus aryabhattai 3	Bacillus simplex 2	Cationic antimicrobial peptide (CAMP) resistance, VraFG transporter	Survival
Bacillus aryabhattai 3	Bacillus simplex 1	Cationic antimicrobial peptide (CAMP) resistance, VraFG transporter	Survival
Arthrobacter oxydans	Bacillus toyonensis 1	gamma-Hexachlorocyclohexane transport system	Extinction
Arthrobacter oxydans	Bacillus toyonensis 2	gamma-Hexachlorocyclohexane transport system	Extinction
Arthrobacter oxydans	Bacillus toyonensis 3	gamma-Hexachlorocyclohexane transport system	Extinction
Arthrobacter oxydans	Cupriavidus basilensis	Fluoroquinolone transport system	Extinction
Arthrobacter oxydans	Bacillus amyloliquefaciens 1	gamma-Hexachlorocyclohexane transport system	Extinction
Arthrobacter oxydans	Bacillus safensis	gamma-Hexachlorocyclohexane transport system	Extinction
Arthrobacter oxydans	Bacillus amyloliquefaciens 2	Adhesin protein transport system	Extinction
Arthrobacter oxydans	Bacillus altitudinis	gamma-Hexachlorocyclohexane transport system	Extinction

Arthrobacter oxydans	Bacillus flexus	Fluoroquinolone transport system	Extinction
Arthrobacter oxydans	Bacillus aryabhattai 1	Fluoroquinolone transport system	Extinction
Arthrobacter oxydans	Arthrobacter humicola	Iron complex transport system	Extinction
Arthrobacter oxydans	Arthrobacter nitroguajacolicus	Iron complex transport system	Extinction
Arthrobacter oxydans	Bacillus aryabhattai 2	Fluoroquinolone transport system	Extinction
Arthrobacter oxydans	Rummeliibacillus pycnus	Fructose transport system	Extinction
Arthrobacter oxydans	Bacillus aryabhattai 3	Iron complex transport system	Survival
Arthrobacter oxydans	Bacillus simplex 2	Capsular polysaccharide transport system	Survival
Arthrobacter oxydans	Bacillus simplex 1	Fluoroquinolone transport system	Survival
Bacillus simplex 2	Bacillus toyonensis 1	gamma-Hexachlorocyclohexane transport system	Extinction
Bacillus simplex 2	Bacillus toyonensis 2	gamma-Hexachlorocyclohexane transport system	Extinction
Bacillus simplex 2	Bacillus toyonensis 3	gamma-Hexachlorocyclohexane transport system	Extinction
Bacillus simplex 2	Cupriavidus basilensis	Cationic antimicrobial peptide (CAMP) resistance, VraFG transporter	Extinction
Bacillus simplex 2	Bacillus amyloliquefaciens 1	gamma-Hexachlorocyclohexane transport system	Extinction

Bacillus simplex 2	Bacillus safensis	gamma-Hexachlorocyclohexane transport system	Extinction
Bacillus simplex 2	Bacillus amyloliquefaciens 2	BceS-BceR (bacitracin transport) two-component regulatory system	Extinction
Bacillus simplex 2	Bacillus altitudinis	gamma-Hexachlorocyclohexane transport system	Extinction
Bacillus simplex 2	Bacillus flexus	Cationic antimicrobial peptide (CAMP) resistance, VraFG transporter	Extinction
Bacillus simplex 2	Bacillus aryabhattai 1	alpha-1,4-Digalacturonate transport system	Extinction
Bacillus simplex 2	Arthrobacter humicola	Iron complex transport system	Extinction
Bacillus simplex 2	Arthrobacter nitroguajacolicus	Fructose transport system	Extinction
Bacillus simplex 2	Bacillus aryabhattai 2	alpha-1,4-Digalacturonate transport system	Extinction
Bacillus simplex 2	Rummeliibacillus pycnus	Cationic antimicrobial peptide (CAMP) resistance, VraFG transporter	Extinction
Bacillus simplex 2	Bacillus aryabhattai 3	Cationic antimicrobial peptide (CAMP) resistance, VraFG transporter	Extinction

Bacillus simplex 2	Arthrobacter oxydans	AlgE-type Mannuronan C-5-Epimerase transport system	Survival
Bacillus simplex 2	Bacillus simplex 1	BceS-BceR (bacitracin transport) two-component regulatory system	Extinction
Bacillus simplex 1	Bacillus toyonensis 1	gamma-Hexachlorocyclohexane transport system	Extinction
Bacillus simplex 1	Bacillus toyonensis 2	gamma-Hexachlorocyclohexane transport system	Extinction
Bacillus simplex 1	Bacillus toyonensis 3	gamma-Hexachlorocyclohexane transport system	Extinction
Bacillus simplex 1	Cupriavidus basilensis	Fructose transport system	Extinction
Bacillus simplex 1	Bacillus amyloliquefaciens 1	gamma-Hexachlorocyclohexane transport system	Extinction
Bacillus simplex 1	Bacillus safensis	gamma-Hexachlorocyclohexane transport system	Extinction
Bacillus simplex 1	Bacillus amyloliquefaciens 2	AlgE-type Mannuronan C-5-Epimerase transport system	Extinction
Bacillus simplex 1	Bacillus altitudinis	gamma-Hexachlorocyclohexane transport system	Extinction

Bacillus simplex 1	Bacillus flexus	Fructose transport system	Extinction
Bacillus simplex 1	Bacillus aryabhattai 1	alpha-1,4-Digalacturonate transport system	Extinction
Bacillus simplex 1	Arthrobacter humicola	Iron complex transport system	Extinction
Bacillus simplex 1	Arthrobacter nitroguajacolicus	Fluoroquinolone transport system	Extinction
Bacillus simplex 1	Bacillus aryabhattai 2	Cationic antimicrobial peptide (CAMP) resistance, VraFG transporter	Extinction
Bacillus simplex 1	Rummeliibacillus pycnus	Fructose transport system	Extinction
Bacillus simplex 1	Bacillus aryabhattai 3	Cationic antimicrobial peptide (CAMP) resistance, VraFG transporter	Extinction
Bacillus simplex 1	Arthrobacter oxydans	Iron complex transport system	Extinction
Bacillus simplex 1	Bacillus simplex 2	AlgE-type Mannuronan C-5-Epimerase transport system	Extinction
